# Fenton-like oxidation–driven dual-mode detection of 2,4,6- trinitrophenyl-N-methylnitramine (tetryl) and 3-nitro-1,2,4-triazole-5-one (NTO) using single-step synthesized Cu_2_O@CuO nanocomposite

**DOI:** 10.1007/s00604-026-08203-3

**Published:** 2026-06-12

**Authors:** Kader Can, Ayşem Üzer, Reşat Apak

**Affiliations:** 1 Department of Chemistry, Faculty of Engineering, Istanbul University-Cerrahpaşa, Avcilar, Istanbul, 34320 Türkiye; 2 Faculty of Pharmacy, Department of Analytical Chemistry, Istinye University, Istanbul, 34010 Türkiye; 3 Turkish Academy of Sciences (TUBA), Bayraktar Neighborhood, Vedat Dalokay St. No:112, Çankaya, Ankara, 06690 Türkiye

**Keywords:** 2,4,6-Trinitrophenyl-N-methylnitramine (tetryl), 3-Nitro-1,2,4-triazol-5-one (NTO), Cu_2_O@CuO nanocomposite, Fenton-like oxidation, Colorimetric detection, Smartphone assisted color detection

## Abstract

A novel detection approach based on single-step synthesized Cu_2_O@CuO nanocomposite (NC) assisted Fenton-like degradation followed by nitrite-mediated colorimetry has been evaluated for the selective separation and determination of 2,4,6-trinitrophenyl-N-methylnitramine (tetryl) and 3-nitro-12,4-triazole-5-one (NTO), which may accompany 2,4,6-trinitrotoluene (TNT) in explosive munitions. Mixed-valent nanocomposite: Cu_2_O@CuONC was successfully synthesized in an alkaline medium without adding a specific reducing agent. The bond cleavage releasing nitrite proceeds differently for the two analytes: ^•^OH radicals, formed as a result of H_2_O_2_ decomposition catalyzed by Cu_2_O@CuONC, leads to the cleavage of the nitramine (N–NO_2_) bond through a non-selective attack on tetryl molecule leading to nitrite elimination, while for NTO molecule, radical species attach to the nitro-substituted C-3 carbon atom in the triazole ring, resulting in simultaneous nitrite elimination. The common major degradation product for tetryl and NTO, i.e. nitrite, reacts with sulfanilamide (SA) in acidic environment to form a diazonium salt, which then reacts with N-(1- naphthyl)ethylenediamine (NED) acting as a coupling agent to form a pink-colored azo dye. The final product could be monitored by both spectrophotometry and a smartphone application. The detection limits for tetryl and NTO were 25.0 µg L^–1^ and 2.0 µg L^–1^ (75.0 µg L^–1^ and 18.0 µg L^–1^ for smartphone application), respectively. The proposed method offers an innovative alternative to the traditional mechanism of forming Meisenheimer complexes for tetryl determination; it is also the first in literature to selectively detect tetryl and NTO, especially in explosive munitions containing TNT.

**Graphical Abstract:**

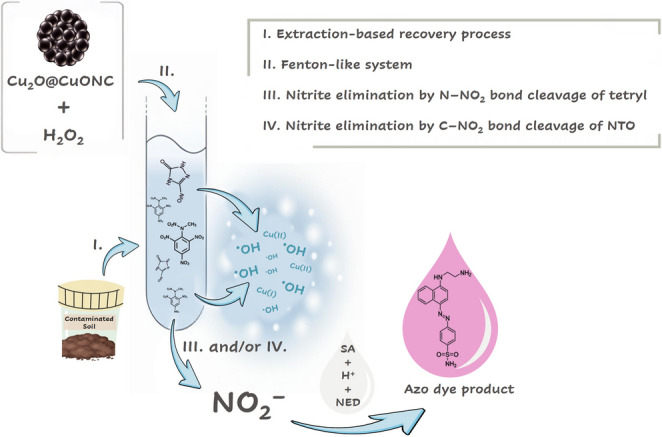

**Supplementary Information:**

The online version contains supplementary material available at 10.1007/s00604-026-08203-3.

## Introduction

During World War II, munition formulations (MFs) containing mixtures of conventional high explosives such as 2,4,6-trinitrotoluene (TNT) and 1,3,5-trinitroperhydro-1,3,5-triazine (Royal Demolition eXplosive, RDX) were developed to enhance their explosive performance [1]. The well-known sensitive explosive formulation of TNT is Tetrytol, which contains both 2,4,6-trinitrophenyl-N-methylnitramine (tetryl) and TNT. Tetryl, an explosive belonging to both nitroaromatic and nitramine classes due to its three aromatic nitro (–NO_2_) groups and one nitramine –NO_2_ group, has been included in sensitive MFs designed in the past because it is a standard booster with a high probability of detonation. This has made it one of the military explosives most likely to be found alongside TNT [2, 3]. On the other hand, in recent years, the US military has been searching for munition that explodes with high performance only when intended and does not explode during transport or storage [4]. The most noteworthy approach proposed for the design of insensitive MFs is the use of less sensitive (i.e., insensitive) explosives in greater proportions relative to sensitive energetics. NTO, 3-nitro-1,2,4-triazole-5-one, having good detonation performance [5], is less sensitive to heat and shock than TNT and RDX, and is therefore a good substitute for these. Thus, insensitive MFs primarily contain an insensitive energetic substance like NTO and, in order to increase explosive performance, a certain proportion of a sensitive conventional explosive such as TNT, RDX, or tetryl, is added [6, 7], meaning that conventional and new-generation nitro-energetics may coexist in sensitive or insensitive MFs [8].

In the literature, almost all techniques (especially colorimetric ones) developed for TNT from nitroaromatics also give a positive analytical signal for tetryl, necessitating their effective separation. Thus, the selective separation and/or determination of tetryl in the presence of TNT in possible formulations where tetryl and TNT may coexist is noteworthy as it fills an important gap in the literature. Due to the high aqueous solubility of NTO (16.6 mg L^− 1^ at 25 °C), and toxic and mutagenic effects of tetryl, selective determination and removal of both nitro-energetics remain an important research topic in terms of both the environmental dimension and building a safer society [4, 9]. Since both compounds have very low vapor pressure, colorimetry is a convenient alternative to criminologic, environmental, and post-blast residue analysis both on-site and in-field. In this regard, simple color tests for the detection of nitro-energetics were first developed under the leadership of Jenkins and Walsh [10], based on the formation of intensely colored Janovsky or Meisenheimer complexes (anionic σ-complexes) of nitroaromatics as a result of the reaction of the electrophilic –NO_2_ groups (electron acceptors) of these energetic compounds with a nucleophilic reagent (electron donor) [11]. On the other hand, the general principle of the colorimetric methods for the determination of nitramines is based on the formation of a colored product with nitrite released as a result of degradation under acidic/basic treatment or ultraviolet irradiation, with a chromogenic reagent. In this context, a dicyclohexylamine (DCHA)-based sensor membrane [12] and potassium hydroxide (KOH)-impregnated microfluidic paper-based analytical devices (µPADs) [13] have been developed for tetryl in the literature. For NTO, there are only a few colorimetric studies based on the formation of yellow Na^+^NTO^–^ salt with NaOH [8, 14, 15] and on phenol-hypochlorite interaction via amine groups [16]. However, using the unique optical and electronic properties of nanomaterials developed in the past two decades, analyses down to the nanomolar level have been successfully performed for various types of explosives, particularly nitro-energetics and other peroxide-type explosives [17–19]. Nanosensors designed to detect explosives with high sensitivity overcome many problems and limitations of conventional detection systems, such as selectivity, size and cost [20, 21]. Therefore, designing nanosensors for detecting nitro-explosives has become a highly reasonable venture [22]. Pioneering studies on this field are colorimetric assays based on the aggregation of gold nanoparticles (AuNPs) enriched with amino (–NH_2_) groups, proposed for the determination of TNT [23, 24]. While colorimetric methods based on aggregation of AuNPs modified with surface functional groups such as –NH_2_, thiol (–SH), and carboxyl (–COOH) groups were developed for tetryl [25–27] and NTO [28], an anti-aggregation-based sensitive colorimetric method with unlabeled AuNPs was reported only for NTO [29].

Copper oxide nanostructures possess unique superior physical and chemical properties such as a high surface-to-volume ratio, superior quantum size effect, high chemical activity, and thermal resistance, making them excellent catalysts in chemical degradation reactions to produce colored products [30]. However, copper oxide nanocomposite (NC) can better host multi-oxidation states of copper, including Cu(0), Cu(I), and Cu(II), offering significant advantages in catalytic processes and sensor applications.

Since most colorimetric assays developed for tetryl and other nitroaromatic analogs are primarily based on the formation of Meisenheimer-type charge-transfer complexes, a selective colorimetric assay of tetryl is absent, and furthermore, a method capable of determining tetryl and NTO via nitrite formation through their –NO_2_ groups has not yet been encountered in the literature. Considering all these factors, this study aimed to selectively detect tetryl (and NTO) using a colorimetric method supported by a nitrite-selective Griess reagent [31] via a Fenton-like degradation process catalyzed by Cu_2_O@CuONC. As a novelty of this system, nitrite-mediated simultaneous colorimetric detection of tetryl and NTO through Fenton-like decomposition process was successfully conducted for the first time in the literature. Compared with the widely used colorimetric methods based on the Meisenheimer-type charge-transfer complexation (for nitroaromatics) and nitrite formation (for nitramines), the system is superior in that it does not respond to TNT and RDX.

## Experimental

### Materials, chemicals and instrumentations

The energetic materials used all over the study, TNT, tetryl, 2,4,6-trinitrophenol (TNP), RDX, 1,3,5,7-tetranitro-1,3,5,7-tetraazacyclooctane (HMX), pentaerythritol tetranitrate (PETN), 1,3,5-trinitrobenzene (TNB) and 4-amino-2,6-dinitrotoluene (4 A-DNT) were graciously supplied by the Mechanical and Chemical Industry Corporation (Makine Kimya Endüstrisi Kurumu-MKEK; Ankara, Turkey) through previous projects. NTO was kindly supplied by Sabancı University (Istanbul, Turkey) as an originally synthesized product from the Chemistry Department’s laboratories. Nitroguanidine (NQ) was obtained from Sigma-Aldrich (St. Louis, Missouri, USA) and ammonium nitrate (NH_4_NO_3_) from Merck (Darmstadt, Germany).

All reagents were analytical grade and used without further purification. All chemicals, materials, and instrumentation used throughout the study are given in the Supplementary Information.

### Preparation of solutions

The preparation of all solutions used throughout the study was described in the Supplementary Information.

### Synthesis of nanomaterials

#### Cu_2_O@CuO nanocomposite

Cu_2_O@CuO nanocomposite (NC) was prepared by adapting the rapid precipitation synthesis procedure reported in the literature [32]. First, 150 mL of 0.02 mol L^–1^ Cu(CH_3_COO)_2_ solution was transferred to a 250 mL Erlenmeyer flask, and then 0.5 mL of glacial acetic acid was added to the solution. The mixture was stirred at 250 rpm with a magnetic stirrer and heated to boiling. Then, 10 mL of 1.0 mol L^–1^ NaOH solution was quickly added to the boiling solution, where a large amount of dark brown precipitate was formed. The Cu_2_O@CuONC in the cooled solution were precipitated by centrifugation to remove excess copper ions, which remained unreacted. Finally, the obtained centrifugates were washed three times with ethanol and allowed to dry in air at room temperature.

#### Magnetite nanoparticles (Fe_3_O_4_NPs)

The synthesis procedure for Fe_3_O_4_NPs is presented in detail in the Supplementary Information.

### Implementation procedure for tetryl and/or NTO indirect determination

A procedure that enables the oxidative degradation of tetryl and/or NTO via a Fenton-like mechanism, followed by colorimetric determination, is described as follows: Firstly, 0.5 mL of a 1000 mg L^–1^ Cu_2_O@CuONC dispersed solution, prepared as a dispersion in an aqueous medium, was transferred to a test tube; 0.5 mL of 1.0 mol L^–1^ H_2_O_2_ and 0.2 mL of 1.0 mol L^–1^ acetic acid–acetate buffer (pH 5.5) were added in sequence. Subsequently, x mL (x ≤ 0.8 mL) of 50.0 mg L^–1^ tetryl and/or NTO standard or soil-extract solution was added and the working solutions, which were completed to a final volume of 2.0 mL with EtOH, were allowed to incubate in a thermostated water bath at 60.0 °C for 40 min. After incubation, 2.0 mL of the Griess reagent, prepared as detailed in the Supplementary Information, was added to the reference and sample solutions, and the pink-colored azo dye formed was measured with a UV-visible spectrophotometer and a smartphone application. The recommended procedure for tetryl and/or NTO samples was schematized in Scheme S1.

### Tests for elucidating the sensing mechanism

In order to investigate the presence of hydroxyl radicals (^•^OH) and superoxide anion radicals (O_2_^•−^) that may be formed as a result of a Fenton-like reaction under Cu_2_O@CuONC catalysis and to evaluate whether these reactive species serve the determination mechanism of the proposed method, terephthalic acid (TA) test [33] for ^•^OH and nitroblue tetrazolium (NBT) test [34] for O_2_^•−^ were applied, as described in the Supplementary Information.

### Selectivity and interference studies

The selectivity and interference study of the proposed method is presented in detail in the Supplementary Information.

### Real sample analysis

Real sample analysis was performed using a certified reference soil sample (CLNSOIL-1) synthetically contaminated with tetryl, NTO and munition samples mixed with TNT in specific mass ratios, that is Tetritol (70% tetryl + 30% TNT) and TNTO (50% NTO + 50% TNT). Clean sandy soil samples, each weighing 2.0 g, were transferred to a series of beakers and contaminated by adding 2.5 mL of tetryl, NTO, Tetritol, and TNTO solutions prepared at 500 mg L^–1^, respectively. The contaminated soil samples were homogenized in an ultrasonic bath and air-dried overnight. After extracting the soil samples contaminated with tetryl and Tetritol with 2.5 mL of ethanol, the soil suspensions were centrifuged (10,000 rpm, 10 min), and the supernatants were filtered using CHROMAFIL GF/PET-45/25, and then the tetryl-containing extracts were diluted to 25.0 mL with ethanol. On the other hand, each soil sample contaminated with NTO and TNTO was treated with 0.50 mol L^–1^ HCl and then homogenized and filtered as described above. Each acidic extract was neutralized with 2.0 and 0.1 mol L^–1^ NaOH (pH ∼ 7) and diluted to a final volume of 25.0 mL with ultrapure water. The proposed method was applied to each extract solution and recovery (%) values for tetryl and NTO were calculated from soil samples contaminated with tetryl, NTO, and their ammunition mixtures. Furthermore, samples containing tetryl and NTO were analyzed using reference LC–MS/MS methods [35, 36] and the results were statistically compared. Experimental details of the reference LC–MS/MS analyses are as described in the Supplementary Information.

### Statistical analysis

The statistical analyses were conducted in Excel (Microsoft Office 2021) software to calculate the mean and standard deviation. Results were reported as the {mean ± standard deviation (SD)}. Method validation against LC–MS/MS determination [35, 36] of tetryl and NTO was assessed using Student’s t- and F-tests for accuracy and precision, respectively.

## Results and discussion

### Synthesis and characterizations of Cu_2_O@CuONC

**Fig. 1:**
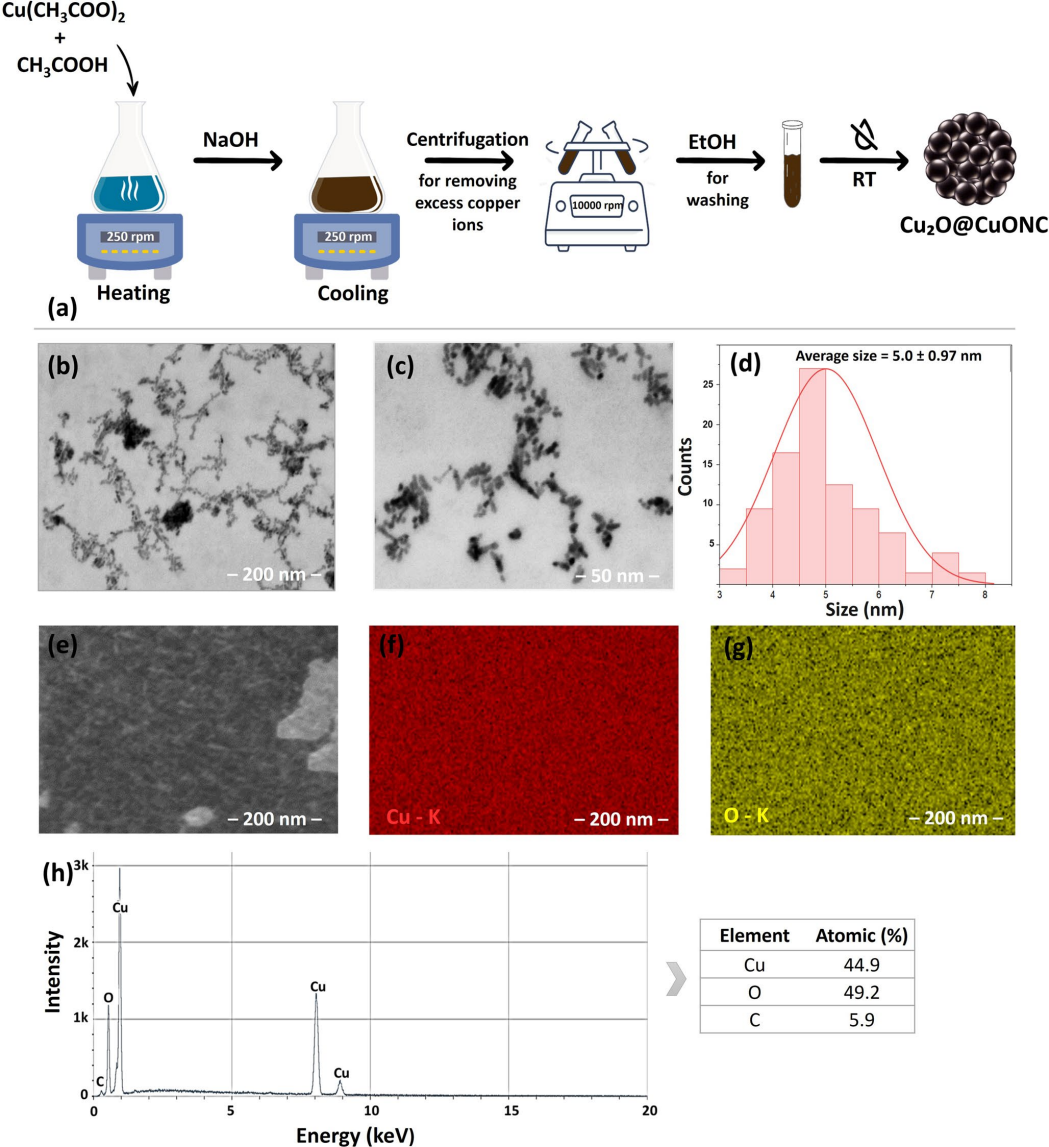
(**a**) Synthesis route of Cu_2_O@CuONC, STEM images of Cu_2_O@CuONC in (**b**) 200 nm and (**c**) 50 nm scale, (**d**) size distribution of Cu_2_O@CuONC using ImageJ software, (**e**–**g**) SEM–EDX mapping images, (**h**) EDX spectrum with the inset showing percentage composition of elements

As seen in Fig. 1, a synthesis route of Cu_2_O@CuONC and a set of characterization studies were conducted to clarify the size and morphology and to confirm the presence of Cu_2_O@CuONC, which were synthesized using the rapid precipitation method in an alkaline medium (Fig. 1a). In STEM images taken at scales of 200 nm (Fig. 1b) and 50 nm (Fig. 1c), it is evident that the synthesized Cu_2_O@CuONC exhibit a spherical morphology with a narrow size distribution. As a result of the evaluation of the STEM images shown in Figs. 1b&c, the average size data of Cu_2_O@CuONC was obtained as 5.0 ± 0.97 nm (Fig. 1d). This confirms that the nanocomposites exhibit a monodisperse distribution in the solution. The SEM-EDX element mapping images (Figs. 1e–g) reflected the homogeneous distribution of the copper (red) and oxygen (yellow) matrix on the NC. Moreover, the EDX spectrum confirmed the presence of copper and oxygen in the NC fraction. The composition of Cu and O elements in the structure of Cu_2_O@CuONC was determined to be 44.9% and 49.2% by atomic percentage, respectively (Fig. 1h). As seen in Fig. 1h, the presence of the C element with an atomic percentage of 5.9% in the structure of Cu_2_O@CuONC can be attributed to the adsorption of a certain amount of C (as CO_2_) from the atmosphere onto the NC surface due to their high surface area/volume ratio.

**Fig. 2:**
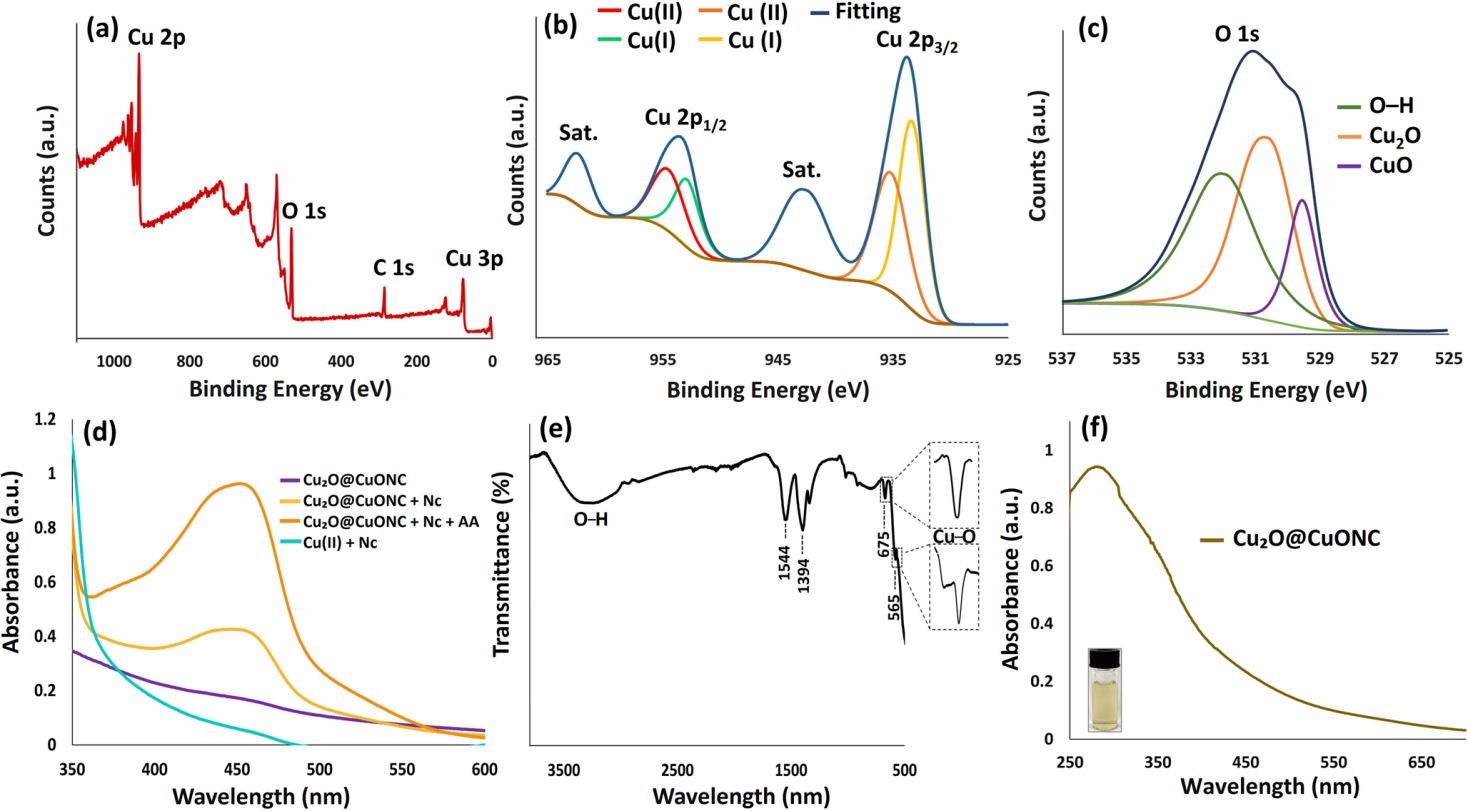
(**a**) XPS survey spectra of Cu_2_O@CuONC, high-resolution spectra of (**b**) Cu 2p, (**c**) O 1 s, (**d**) UV−visible absorption spectra of different reaction systems, (**e**) ATR-FTIR spectra and (**f**) UV−visible absorption spectra of Cu_2_O@CuONC, with the inset showing a vial image of the dispersed solution of Cu_2_O@CuONC

The verification of the element analysis and surface composition of Cu_2_O@CuONC was carried out by XPS analysis, as shown in Figs. 2a–c. The XPS survey spectrum of the Cu_2_O@CuONC suggests that only Cu, O, and C (from atmospheric) elements were detected and no other impurities were observed (Fig. 2a), which is consistent with the SEM-EDX results. Fig. 2b suggests the Cu 2p high-resolution XPS spectrum, including the Cu 2p_1/2_ and Cu 2p_3/2_ peaks with two satellite peaks. The fitting peaks for Cu 2p_1/2_ and Cu 2p_3/2_ correspond to nearly 953.4 eV and 933.5 eV, respectively. Two peaks located at 952.9 and 933.7 in the Cu 2p_1/2_ and Cu 2p_3/2_ XPS spectra, respectively, are attributed to Cu(I) species, while the peaks located at 954.8 and 935.1 correspond to Cu(II) species [37, 38]. The observation of Cu(I) and Cu(II) peaks indicates that the overall composition of the NC consists of both Cu_2_O and CuO phases. Of note is the fact that copper can exist in both divalent and monovalent valences with 3d^9^ and 3d^10^ electron-configurations, respectively [39]. As seen in Fig. 2b, the characteristic satellite peaks centred at high binding energies of 962.3 eV and 942.5 eV are evidences of the 3d^9^ configuration, further supporting the presence of Cu(II) corresponding to the CuO phase [39, 40]. As shown in Fig. 2c, the O 1 s high-resolution XPS spectrum includes three peaks. These peaks, centred at 530.7 and 529.5 eV, can be attributed to oxygen in the Cu_2_O and CuO lattices, respectively. Meanwhile, the peak with a higher binding energy of 531.9 eV may originate from the O–H peak adsorbed on the Cu_2_O@CuONC lattice [39, 41, 42].

To confirm the oxidation states of copper in the NC, a colorimetric assay was also performed using the chromogenic reagent neocuproine (Nc), a strong chelating agent selective for cuprous ions [43]. In Fig. 2d, the UV-visible spectra of the Cu_2_O@CuONC, Cu_2_O@CuONC + Nc, Cu_2_O@CuONC + Nc + ascorbic acid (AA), and Cu(II) + Nc systems are presented, respectively. When the dispersed Cu_2_O@CuONC solution reacts with Nc, the Cu(I) ions in the Cu_2_O@CuONC form a yellow Cu(I)-Nc chelate, which produces a characteristic peak at 452 nm, suggesting that Cu(I) ions are present in the NC. Furthermore, when ascorbic acid is added as a reducing agent to the system containing Cu_2_O@CuONC and Nc, the signal at 452 nm increases significantly. This suggests that the Cu(II) ions in the NC are reduced to Cu(I) ions by AA [44]. As opposed to this, no significant signal belonging to Cu(I)-Nc chromogen appears in the UV-visible absorption spectra of the dispersed Cu_2_O@CuONC and Cu(II) + Nc system. All results corroborate that the surface of the Cu_2_O@CuONC contains a mixture of Cu(I) and Cu(II) in different valencies.

Based on the collective evidence above, the findings were quite surprising. Under high temperatures and basic conditions (pH ≥ 10), Cu(II) acetate rapidly hydrolyzes to form Cu(OH)_2_, and the resulting hydroxide phase is converted to CuO through thermal dehydration during heat treatment [32]. However, the reduction of Cu(II) to Cu(I) to form the Cu_2_O phase without any reducing agent is not an expected outcome. In other words, the reduction of Cu (II) to Cu (I) is a reduction reaction that generally requires an electron source. It is known that the actual O_2_/H_2_O reduction potential (O_2_ + 4e^–^ + 4 H^+^ ⇋ 2H_2_O, E_O2/H2O_), which is 1.23 V vs. normal hydrogen electrode (NHE) under standard conditions, decreases under alkaline conditions [45]. Also, Zheng et al. (2014) reported that the standard reduction potential of the CuO/Cu_2_O is 0.60 V vs. NHE [46]. The fact that E_O2/H2O_ is close to the boundary of the CuO/Cu_2_O potential under alkaline conditions indicates that the electron source for the reduction of Cu (II) to Cu (I) can be provided via the oxygen evolution reaction (OER). In the literature, it has been reported that Cu(II)-oxo species can be converted to Cu(I) at high temperatures without a reducing agent, and that this autoreduction is achieved with the release of oxygen [47]. This finding suggests that Cu_2_O is more stable than CuO in aqueous alkaline solution and is least soluble among copper oxides, indicating that the equilibrium favors Cu_2_O [45, 47]. Given this information, under the conditions of NC synthesis, changes in the OER potential with pH may have enabled the non-stoichiometric formation of CuO and Cu_2_O phases by self-reduction.

Cu_2_O@CuONC were analysed using ATR-FTIR spectroscopy in the 400–4000 cm^–1^ range, as shown in Fig. 2e. The characteristic peaks at 675 cm⁻¹ and 565 cm⁻¹ vibration bands can be attributed to Cu(I)–O and Cu(II)–O stretching vibrations, respectively [48], confirming the presence of Cu–O bonds, and consequently the successful synthesis of Cu_2_O@CuONC. The broad band observed in the FTIR spectrum between 3630 cm^1^ and 2960 cm^–1^ corresponds to the –OH stretching vibration of water molecules absorbed as a result of moisture absorption in the lattices due to the high surface area/volume ratio of Cu_2_O@CuONC. The peaks observed at 1544 cm^–1^ and 1394 cm^–1^ are attributed to the C = O stretching vibrations of the carboxylate ion, which is bound as a bidentate ligand to the Cu_2_O@CuONC synthesized in the acetate matrix [49]. Moreover, the peak at 1544 cm^–1^ also reflects the formation of a covalent bond between the –OH and –COOH groups on the surface of the Cu_2_O@CuONC, forming the (–C(= O)–O–Cu) [49].

The synthesized Cu_2_O@CuONC exhibited good dispersion in ultrapure water, forming a transparent brown solution (inset of Fig. 2f). The UV-visible absorption spectra are represented in Fig. 2f, where the characteristic absorption peak of Cu_2_O@CuONC was obtained at 280 nm.

### Design and optimization of Fenton-like degradation system

The proposed method for the determination of tetryl and NTO, based on metal oxide-catalyzed decomposition followed by nitrite-mediated colorimetry, requires thorough investigation of certain parameters that are critical in terms of analytical performance of the system. Certain parameters in the optimization studies were investigated in the presence of the representative analyte, tetryl. In this context, the selection of a metal oxide-based nanomaterial type to be used as a catalyst in a Fenton-like reaction was first examined. The nanostructures Fe_3_O_4_NPs and Cu_2_O@CuONC were synthesized from iron and copper sources, and experiments were conducted under the same experimental conditions for both metal oxide nanostructures. Their catalytic activities were compared using visible absorption spectra and bar diagrams (Fig. S1). Although the system was not optimized at this stage, the operating pH of the proposed method is quite critical in terms of the major degradation product of tetryl (i.e., nitrite). This is because, as a result of the Fenton-like degradation process of tetryl, the relevant degradation product nitrite is converted into species that are predominant in the significantly acidic region (pH < 3.0); in other words, it is converted into nitrous acid (HONO) and then into its protonated (H_2_ONO⁺) form, that may result in its losses in the form of nitrogen oxides [50]. Therefore, preliminary tests were conducted to identify a suitable catalyst for the proposed method under pH 5.5 reaction conditions, with a focus on minimizing nitrite loss. Evaluation of results showed that Cu_2_O@CuONC exhibit significantly superior catalytic performance compared to Fe_3_O_4_NPs for the proposed method (Fig. S1). This situation may be explained by the fact that Fe(II) and Fe(III) ions in Fe_3_O_4_NPs exist in different forms depending on pH, thereby decreasing the concentration of free iron ions and consequently limiting the formation of radical species. The optimal reaction pH value for the traditional iron-based Fenton reaction is between 2.0 and 4.0 [51]. In this pH range, iron ions maximise radical production by increasing the decomposition of H_2_O_2_. However, when the reaction pH is above 4.0 (pH > 4.0), the activity of the Fe_3_O_4_NP-based Fenton reaction decreases due to the formation of hydrolytic Fe(II) complexes {i.e., Fe(OH)^+^} and the precipitation of Fe(III) hydroxides {i.e., Fe(OH)_3_} [52]. This phenomenon, which directly affects the degradation efficiency of tetryl to nitrite, is considered the primary reason for the significant decrease in the analytical signal obtained with Griess reagent *via* Fe_3_O_4_NPs catalysis compared to Cu_2_O@CuONC catalysis. In summary, the decrease in the free iron source at pH 5.5 limits the formation of other possible radical species, primarily the reactive ^•^OH species formed as a result of H_2_O_2_ decomposition in Fe_3_O_4_NPs catalysis, leading to a decrease in the analytical signal obtained from the tetryl degradation product, nitrite (Fig. S1). On the other hand, copper-based Fenton-like catalysts in generating radical species (especially ^•^OH) are efficient at neutral or near-neutral conditions [51] and the operating pH of the proposed method (i.e., pH 5.5) is within the ideal pH range for demonstrating the catalytic performance of Cu_2_O@CuONC. Consequently, Cu_2_O@CuONC were selected as a suitable catalyst for all subsequent experiments. Furthermore, the superior catalytic properties of Cu_2_O@CuONC were compared with the aqueous solution of the salt used as the Cu(II) source in the synthesis of the NC, to reveal that Cu_2_O@CuONC exhibited higher catalytic performance compared to the Cu(II) salt (Fig. S1). This is attributed to the nanoscale size and superior surface properties of Cu_2_O@CuONC and the mixed-valence nature of the Cu_2_O@CuONC lattice [48, 53]. As shown in Fig. S2, after determining the appropriate catalyst, experiments were conducted at an initial concentration range of 500–3000 mg L^─1^ and the initial Cu_2_O@CuONC concentration, at which the maximal absorbance was obtained at 542 nm, was determined as 1000 mg L^─1^.

The optimal initial concentration of H_2_O_2_, acting as an oxidant in the Fenton-like reaction system, for maximal oxidation efficiency was determined as 1.0 mol L^─1^ (Fig. S3). As shown in Fig. S3, a sudden drop in Griess absorbance intensity at 542 nm was seen at H_2_O_2_ concentrations above 1.0 mol L^─1^. This is attributed to excess H_2_O_2_ in the medium, which reacts with the ^•^OH formed by the Fenton-like oxidation process, acting as a free radical scavenger, reducing the concentration of ^•^OH and producing less reactive hydroperoxyl radicals (^•^OOHs) [54]. This significantly reduced the decomposition efficiency of tetryl (Fig. S3).

As shown in Fig. S4, the effect of Cu_2_O@CuONC: H_2_O_2_ (v/v) ratio on the catalytic degradation of the representative analyte (i.e., tetryl) was examined during a Fenton-like oxidation process, where the maximal reaction efficiency was achieved at 1:1 Cu_2_O@CuONC: H_2_O_2_ (v/v) ratio, corresponding to a final Cu_2_O@CuONC concentration of 125 mg L^− 1^ in the reaction medium (Fig. S4). The decrease in analytical signals obtained at high Cu_2_O@CuONC or H_2_O_2_ amount is attributed to the scavenging of ^•^OH by excess reaction initiators {here Cu(I)/Cu(II) and H_2_O_2_} present in the medium [54, 55] and/or to a decrease in the presence of ^•^OH due to the excessive formation of undesirable byproducts of oxidation.

As noted above, due to pH-dependent nitrite speciation, experiments were carried out within the pH range between 4.0 and 7.0 (Fig S5). As shown in Fig. S5, the maximum absorbance value of the pink azo dye at 542 nm formed by the reaction of nitrite, the degradation product of the Cu_2_O@CuONC-based Fenton-like reaction, with Griess reagent was observed at pH 5.5 in acetic acid/acetate buffer medium. The literature suggests that the acetic acid–acetate buffer system offers optimal oxidation efficiency in the Cu-driven Fenton-based reaction, whereas phosphate and sulfate buffers are less effective [56]. Additionally, Cu_2_O@CuONC tends to dissociate into copper ions at pH 5.5 in acetate buffer, which increases their catalytic activity [57].

Incubation temperature and time experiments for the degradation of the representative analyte in the Fenton-like reaction were performed at 45–70 °C and 10–60 min, respectively. Maximal analytical signals were obtained after incubation at 60 °C for 40-min as shown in Fig. S6 and Fig. S7, respectively.

### Elucidating the plausible sensing mechanism

**Fig. 3:**
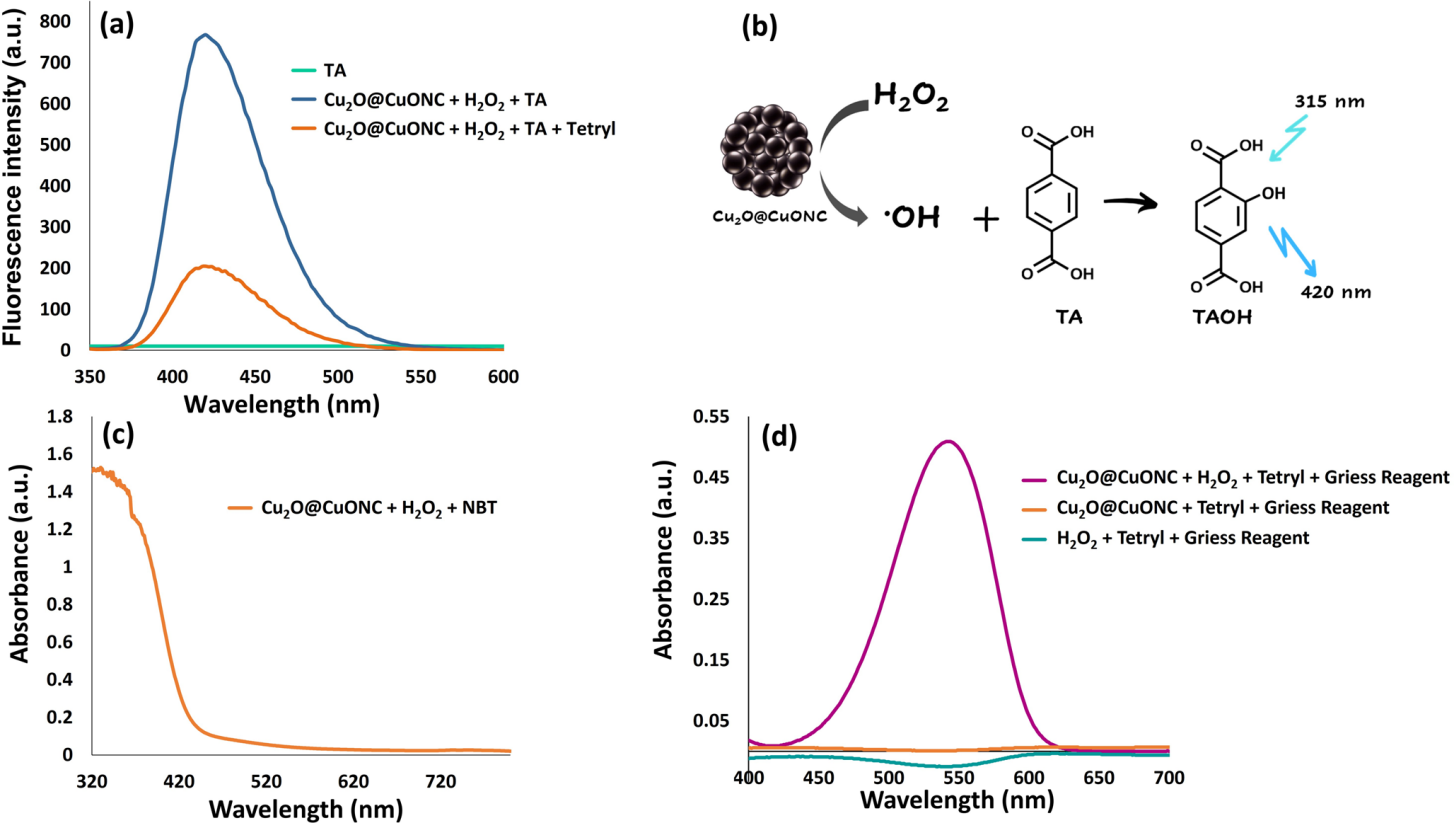
(**a**) Fluorescence spectra of TA, Cu_2_O@CuONC + H_2_O_2_ + TA and Cu_2_O@CuONC + H_2_O_2_ + TA + tetryl systems, (**b**) schematic illustration of the test for the production of hydroxyl radicals using the proposed Fenton-like system, (**c**) UV−visible absorption spectra of the Cu_2_O@CuONC + H_2_O_2_ + NBT system (**d**) UV−visible absorption spectra obtained by applying the Griess reagent after the decomposition of tetryl in the presence of Cu_2_O@CuONC + H_2_O_2_, Cu_2_O@CuONC and H_2_O_2_ systems

Determining the generation and consumption of reactive oxygen species (ROS), such as ^•^OH and O_2_^•−^, likely to form in a Fenton-like reaction, is extremely important for elucidating the mechanism of the proposed method. In this context, some colorimetric and fluorometric tests, as detailed in the Supplementary Information, were employed. Firstly, a fluorogenic probe: terephthalic acid (TA) was used to determine ^•^OH, the primary reactive species expected to form in a Fenton-like reaction, and to prove that these reactive species are consumed by tetryl. TA reacts with free ^•^OH in the medium to form the fluorescent hydroxyterephthalatic acid (TAOH) [33]. As shown in Fig. 3a, no fluorescence was observed with TA alone, whereas a strong fluorescence signal at a wavelength of 420 nm was obtained in the Cu_2_O@CuONC + H_2_O_2_ + TA system. This signal indicates that ^•^OH formed from the Cu_2_O@CuONC−catalyzed decomposition of H_2_O_2_ reacts with TA to form the fluorescent product TAOH (as shown in Fig. 3b). This supports the formation of free ^•^OH in the reaction system, indicating the peroxidase-like activity of Cu_2_O@CuONC to catalyze the decomposition of H_2_O_2_ into ^•^OH. As shown in Fig. 3a, a significant decrease in fluorescence intensity was observed in the Cu_2_O@CuONC + H_2_O_2_ + TA system in the presence of tetryl. This indicates that the ^•^OH producing TAOH from TA is consumed during the decomposition of tetryl. In order to detect O_2_^•−^, the spectrophotometric nitroblue tetrazolium (NBT) test [34] was employed. The pale yellow colored chromogenic reagent NBT in ethanol transforms into a blue formazan species, a reduced form of NBT, in the presence of O_2_^•−^, giving a characteristic absorbance signal at approximately 560 nm [58]. Under the tested conditions, the spectrum shown in Fig. 3c clearly shows that O_2_^•−^ was not detected in the Cu_2_O@CuONC + H_2_O_2_ + NBT system, indicating that O_2_^•−^ is not a product of the system.

To evaluate the efficiency of the Cu_2_O@CuONC-catalyzed Fenton-like reaction developed for the degradation of target analyte(s) and to support the elucidation of the mechanism, tetryl solutions were subjected to the same degradation process in the presence of Cu_2_O@CuONC and H_2_O_2_, Cu_2_O@CuONC alone, and H_2_O_2_ alone. After degradation, the Griess reagent (namely, a strongly acidic solution of a mixture of SA and NED) was added to the cooled samples, and visible spectra for each variable were obtained (Fig. 3d). Fig. 3d shows that the decomposition product was Griess-positive. This clearly demonstrates the presence of nitrite among the primary decomposition product(s) in tetryl decomposition. The assumed mechanism is given in the Supporting Information as “Hypothetical Reaction Mechanism for -N(CH_3_)-NO_2_ Bond Cleavage to Produce Nitrite” (S4), since highly reactive intermediates were not identified. The reaction of the released nitrite with the Griess reagent resulted in a pink-colored azo dye with maximal absorbance at 542 nm. As seen in Fig. 3d, the absence of an analytical signal in the presence of the Griess reagent during the degradation of tetryl with either Cu_2_O@CuONC alone or H_2_O_2_ alone proves that nitrite formation is realized by ^•^OH generated with a Fenton-like reaction in the presence of both Cu_2_O@CuONC and H_2_O_2_.

**Scheme 1:**
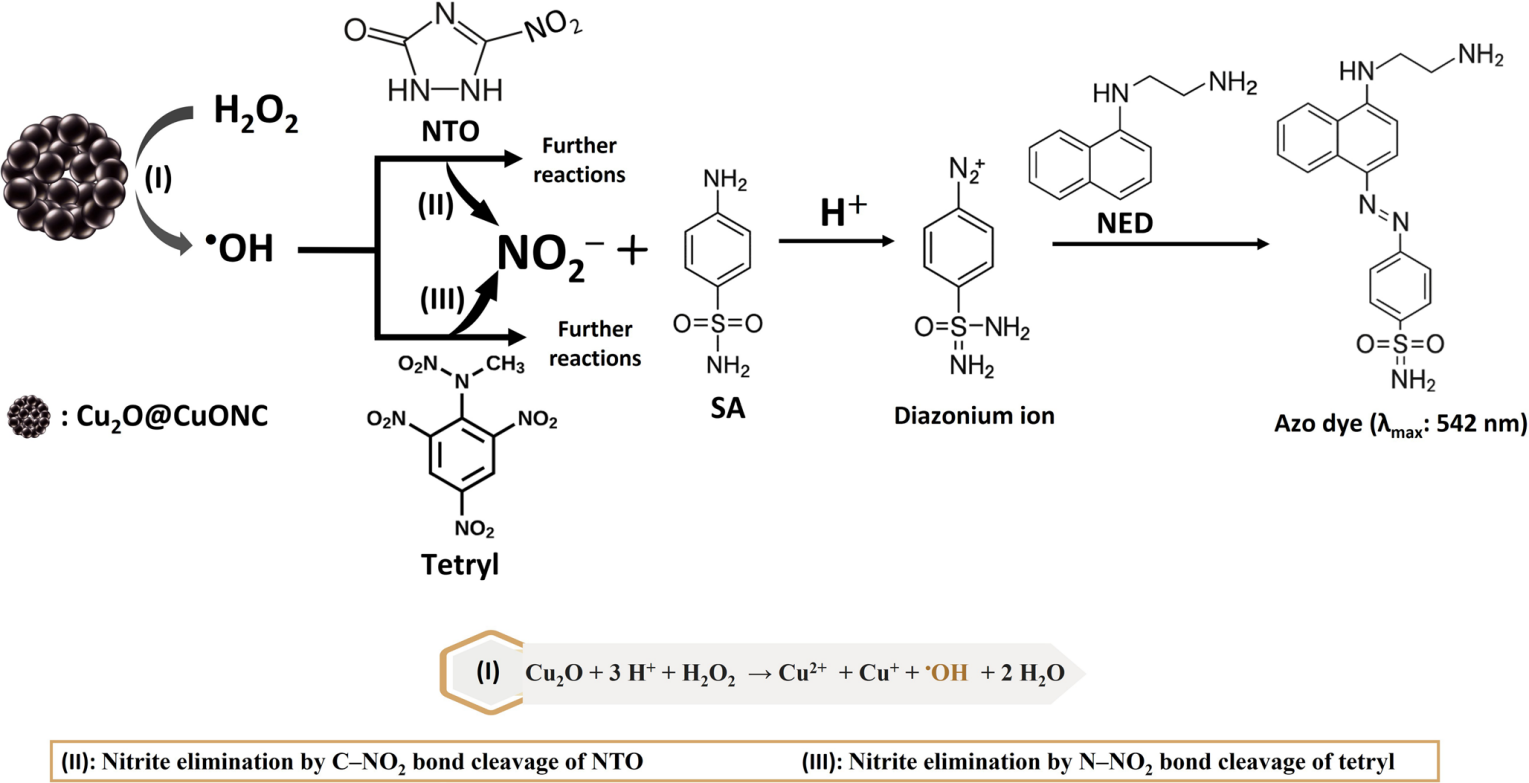
Schematic illustration of the primary nitrite elimination occurring in the Fenton-like degradation of tetryl and NTO [61] and the possible reaction mechanism of the released nitrite in the presence of Griess reagent

Based on the collective evidence above, this mechanism encompasses three interconnected pathways. Schematic representations of the possible reaction mechanism, involving Path-1 (Fenton-like reaction): formation of reactive ^•^OH as a result of the decomposition of H_2_O_2_ catalyzed by Cu_2_O@CuONC; Path-2 (degradation of target analyte(s)): degradation of the target analytes via the generated ^•^OH; Path-3 (Griess reaction): the formation of a diazonium salt with the resulting decomposition product nitrite under acidic conditions with SA and then the formation of an azo dye with NED as a coupling agent [31], is shown in Scheme 1. To elaborate on Path-2 for both analytes, firstly, as seen in Scheme 1, tetryl is an explosive classified as both nitroaromatic and nitramine due to the presence of nitro (− NO_2_) functional groups on the aromatic ring and nitramine (− N−NO_2_) functional group outside the ring [2]. The −NO_2_ groups, which are strongly electron-withdrawing, deactivate the aromatic ring by reducing its electron density, while the methyl(nitro)amine (− NCH_3_NO_2_) group outside the ring is an electron donor, increasing the electron density of the aromatic ring, strengthening the resonance stability of the structure and activating the ring [59]. However, the electron donation of the −NCH_3_NO_2_ group to the aromatic ring weakens the N−NO_2_ bond by making it electron-poor [2]. Thus, it is thought that the cleavage of the N−NO_2_ bond, which is relatively straightforward to break releasing nitrite, plays an active role as the first step in the decomposition of tetryl in the presence of ^•^OH. To support this idea, the bond dissociation energies (BDEs) of the functional groups of tetryl were examined in the literature. Tan et al. (2010) calculated the BDEs of the X−NO_2_ (X = C, N, and O) groups of explosives using various methods based on quantum chemistry and reported the BDE of the N−NO_2_ group of tetryl as 134.6 kJ mol^–1^. Furthermore, the study reported that the BDEs of the C−NO_2_ groups of nitroaromatic explosives, which are tetryl derivatives, ranged from approximately 260 to 278 kJ mol^–1^ [60]. These results clearly show that the N−NO_2_ bond, which constitutes the nitramine of tetryl, is considerably weaker than the C−NO_2_ bond. In conclusion, the theoretical approaches given above regarding nitrite release from tetryl support the idea that •OH produced in the reaction system non-selectively attacks the functional groups of tetryl, leading to the cleavage of the N−NO_2_ bond, which is more susceptible to radical attack, as the first step of degradation and the release of the primary cleavage product, nitrite.

The study reported by Le Campion et al. (1998) has been a significant source in shaping the degradation mechanism of NTO through the Fenton-like oxidation process [61]. Based on this mechanism, the degradation of anionic form of NTO *via* Fenton-like oxidation catalyzed by Cu_2_O@CuONC, and the possible reaction mechanism for the formation of a pink-colored azo dye as a result of the reaction of the degradation byproduct nitrite with Griess reagent are shown in Scheme 1. It is notewothy that the decomposition reaction scenario of anionic NTO (pK_a_ for NTO: 3.76) by ^•^OH at the working pH (pH 5.5) was reported to be similar to that of the neutral NTO form [62]. As shown in Scheme 1, nitrite elimination occurs simultaneously with the addition of ^•^OH to the nitro-substituted C-3 carbon atom in the triazole ring of the NTO molecule.

### Analytical performance of sensing system

#### Sensitivity of the proposed method

**Fig. 4:**
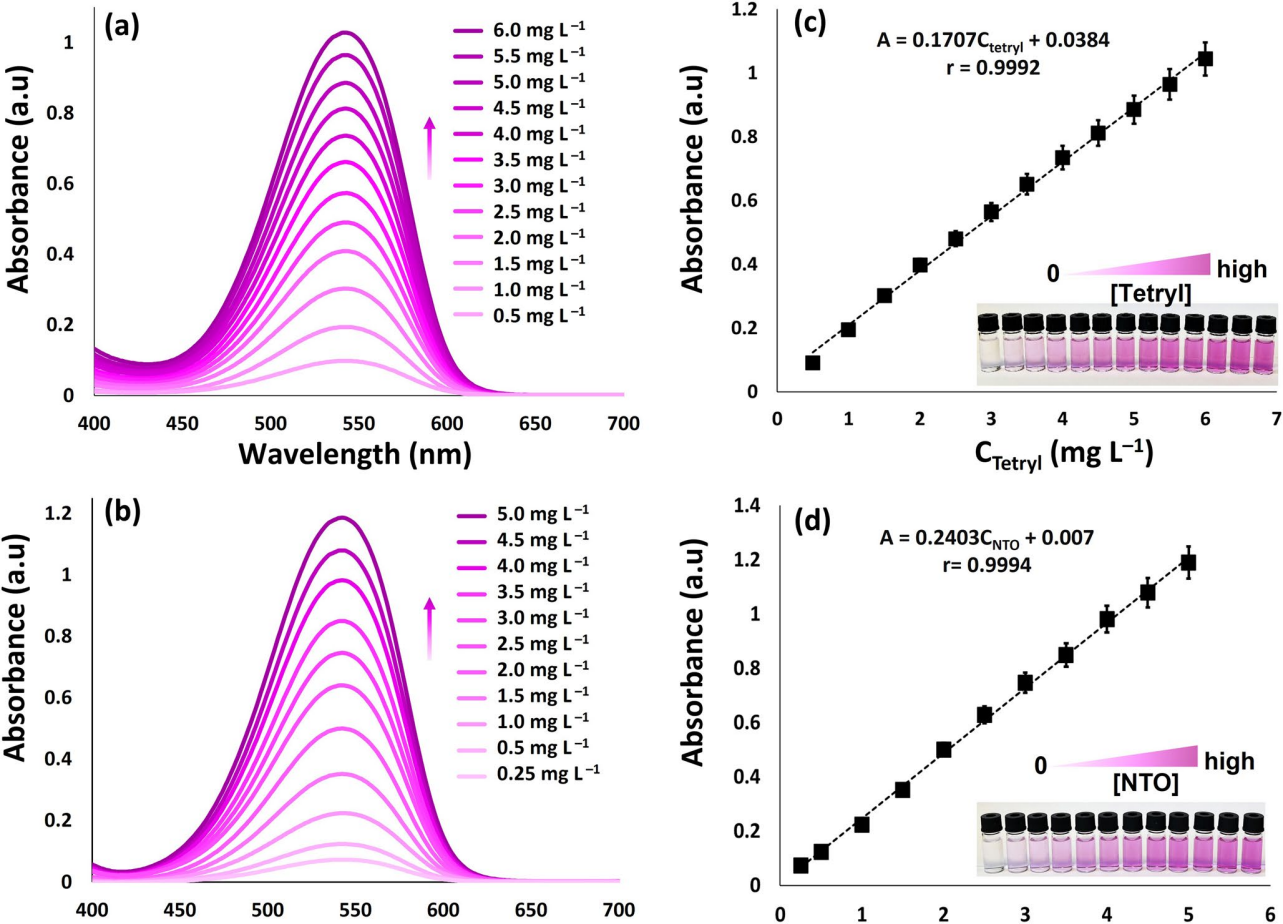
(**a**) UV−visible absorption spectra obtained by applying the Griess reagent after the decomposition of tetryl (0.50–6.0 mg L^─1^, final conc.) solutions by the proposed Fenton-like oxidation, (**b**) plot of the absorbance at λ_max_ = 452 nm as a function of the final concentration of tetryl (0.50–6.0 mg L^─1^, final conc.) with the image of corresponding test tubes (inset), (**c**) UV−visible absorption spectra obtained by applying the Griess reagent after the decomposition of NTO (0.25–5.0 mg L^─1^, final conc.) solutions by the proposed Fenton-like oxidation, and (**d**) plot of the absorbance at λ_max_ = 452 nm as a function of the final concentration of NTO (0.25–5.0 mg L^─1^, final conc.) with the image of corresponding test tubes (inset)

To investigate the sensitivity of the proposed method, the visible spectra of the pink-colored azo dye formed from the reaction of tetryl- and NTO-derived nitrite with Griess reagent, linear calibration curves with calibration equations and color changes (as inset image of test tubes) are shown in Fig. 4.

The analytical signals (*N* = 3) obtained under optimized conditions are linearly related to increasing concentrations of the target analytes, both tetryl and NTO. According to Fig. 4a, the absorption signal at 542 nm (measured against a blank) gradually increased as the tetryl concentration was varied from 0.5 to 6.0 mg L^− 1^ and a calibration curve was obtained by plotting the resulting absorbance values against the tetryl concentration (Fig. 4b), displaying excellent linearity within the indicated range (see Eq. 1):

1 $$\:{\:\mathrm{A}}_{452}=0.1707\:{\mathrm{C}}_{\mathrm{t}\mathrm{e}\mathrm{t}\mathrm{r}\mathrm{y}\mathrm{l}}+0.0384\:(\mathrm{r}=0.9992)$$

where A_452_ is the absorbance value at 452 nm and C_tetryl_ is the final concentration of tetryl in mg L^–1^.

As shown in Fig. 4c, the absorption signal at 542 nm gradually increased with increasing NTO concentration (0.25–5.0 mg L^− 1^) with good linearity of the calibration curve (Fig. 4d) (see Eq. 2):

2 $$\:{\mathrm{A}}_{452}=0.2403\:{\mathrm{C}}_{\mathrm{N}\mathrm{T}\mathrm{O}}+0.007\:(\mathrm{r}=0.9994)$$

where A_452_ is the absorbance value at 452 nm and C_NTO_ is the final concentration of NTO in mg L^–1^.

The analytical performance parameters of the developed method for the determination of tetryl and NTO are summarized in Table 1.

**Table 1:** Analytical performance parameters of the developed method for tetryl and NTO

Analyte	Linear range^a^	LOD^b^	RSD^c^ (%)	
			intra-assay	inter-assay
Tetryl	0.50–6.0	25.0	0.37	0.76
NTO	0.25–5.0	2.0	0.32	0.67

^a^In mg L^–1^ units (final conc.), ^b^Limit of detection, in µg L^–1^ units (LOD = 3σ_bl_/m, σ_bl_ denoting the standard deviation of a blank, and m showing the slope of the calibration line) and ^c^Relative standard deviation, as percentage (*N* = 5)

#### Evaluation of selectivity and possible interference effects

**Fig. 5:**
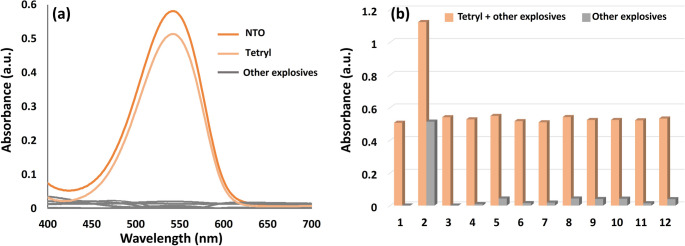
(**a**) Comparison of the visible spectra obtained from the proposed Griess reagent on Fenton-like oxidation driven system in the presence of tetryl, NTO and other potential interfering energetic compounds and (**b**) absorbance intensity of Griess reagent on Fenton-like oxidation driven system in the presence of tetryl (orange bars) and in the absence of tetryl (gray bars) with different types of energetic materials (NTO 1-, TNT 50- and the others 10-fold): tetryl (only) (1), NTO (2), NTO after extraction (3), TNT (4), TNP (5), RDX (6), HMX (7), PETN (8), TNB (9), 4 A-DNT (10), NQ (11), and NH_4_NO_3_ (12)

To determine the selectivity of the proposed method, the potential effects of other energetic compounds (NTO, TNT, TNP, RDX, HMX, PETN, TNB, 4-ADNT, NQ, and NH_4_NO_3_) on the method were investigated in the presence and absence of tetryl. For this purpose, the proposed method was applied to synthetically prepared above-mentioned energetic compounds at 1- and 10-fold (1-fold for NTO and 1-, 10-, and 50-fold for TNT) concentrations in the presence of tetryl (2.5 mg L^–1^, final conc.), and the recovery values of tetryl were calculated as a percentage (%). As shown in Table S1, the recoveries of tetryl from binary mixtures of (tetryl + energetic compound) ranged from 100.7% to 108.3%. The UV-vis absorption spectra obtained by applying the proposed method to tetryl, NTO and other energetic compounds and a bar diagram of interference effects showing the recoveries of the representative analyte tetryl, both alone and in admixtures with NTO and other energetic compounds are shown in Figs. 5a&b. As seen in Fig. 5, it was clear that azo dye formation resulted only from tetryl and NTO, and no significant absorbance response was obtained from other energetic compounds. To effectively separate NTO from the tetryl–NTO binary synthetic mixture, a solubility difference-based separation technique was proposed, exploiting the preferential aqueous solubility of NTO over tetryl (tetryl solubility in water is 0.008 g L^− 1^/25 °C; NTO solubility in water is 16.6 g L^− 1^/25 °C) [63, 64], the details of which are given in the Supplementary Information.

Reports in the literature describe the decomposition of explosives via Fenton or Fenton-like oxidation processes [59, 65, 66]. Since tetryl is an explosive belonging to both the nitroaromatic and nitramine classes [2], literature reviewers have focused on TNT, representing the nitroaromatic class, and RDX, representing the nitramine class. Firstly, different mechanism scenarios for the decomposition of TNT via Fenton oxidation have been suggested [59, 65, 66]. Li et al. (1997) reported that the final decomposition products are NO_3_^–^, CO_2_, and H_2_O via a two-pathway mechanism involving the oxidation and decarboxylation of the methyl (–CH_3_) group of TNT [67]. In a study by Matta et al. (2007), it was reported that the oxidative decomposition of TNT proceeds in two different pathways after the addition of ^•^OH to the ring by attacking the methyl-substituted C-1 carbon atom. The first pathway involves the dimerization of two hydroxylated structures, while the second pathway involves the formation of epoxides that trigger ring opening, followed by the formation of intermediates where ring hydroxylation continues. It has been reported that oxalic acid (H_2_C_2_O_4_), formic acid (HCOOH), and NO_3_^–^ are released as end products of both decomposition mechanisms [66]. Consequently, it is evident that nitrite is not formed as a decomposition product in both studies that propose different mechanisms for the Fenton-based oxidative degradation of TNT. Similarly, it is clear from Fig. 5 that other energetic materials of the nitroaromatic class (TNP, TNB, 4-ADNT) besides TNT do not respond to the system. These results also support the idea that nitrite, the decomposition product of the Fenton-like oxidation of tetryl, arises from the cleavage of the N–NO_2_ bond outside the ring. On the other hand, it is important to examine the interference effect of RDX, a heterocyclic nitramine containing multiple N–NO_2_ functional groups. Dai-Lam et al. (2014) studied the decomposition efficiencies of tetryl and RDX, which are both nitramines and compared them using advanced oxidation processes (electro-oxidation and Fenton oxidation). The authors found that RDX decomposed with a lower efficiency compared to tetryl, attributing this to the difference in the molecular structures of RDX and tetryl, belonging to heterocyclic nitramine and nitroaromatic nitramine classes, respectively. RDX is more resistant to possible oxidative attacks, and consequently, under the same conditions, RDX decomposed to a lower extent than tetryl [68]. The BED values of the N–NO_2_ group of RDX were investigated, and it was found to be approximately 200.8 kJ mol^–1^, indicating that primary decomposition in oxidation processes occurs via cleavage of the N–NO_2_ bond at this energy level [65]. This value corresponds to a considerably higher energy barrier compared to the 134.6 kJ mol^–1^ BED value of the N–NO_2_ bond of tetryl [60], suggesting that breaking the N–NO_2_ bond of tetryl is thermodynamically more favorable in an oxidative degradation reaction. The experimental findings (as seen in Table S1) revealed that nitrite was not released as a decomposition product during the proposed oxidation process of RDX, meaning that the proposed decomposition process does not reach the energy threshold required for RDX to overcome the BED barrier of the N–NO_2_ bond. Figs. 5a&b clearly show that the possible decomposition products of different classes of explosive materials (nitroaromatics, nitramines, nitrate esters, and inorganic explosives) do not interfere with the determination mechanism of the proposed method (i.e., they do not produce nitrite), explaining the selectivity of the method for tetryl and NTO over other similar explosives.

To evaluate the applicability of the proposed method, the effects of certain ions frequently encountered in environmental systems (Na^+^, K^+^, Ca^2+^, Mg^2+^, Fe(II), Fe(III), Al^3+^, Cl^−^, NO_3_^−^, CO_3_^2−^ and SO_4_^2−^) and camouflage materials used during the transport of energetic materials (acetylsalicylic acid (aspirin), aspartame, household detergent, ᴅ-(+)-glucose and paracetamol) on the proposed method were investigated. For this purpose, the proposed method was first applied to synthetic mixture solutions containing ionic species prepared at concentrations of 1-, 10- and 100-fold by mass (1-, 10- and 50-fold for Al^3+^ and CO_3_^2–^) compared to tetryl. According to the data obtained in Table S2, the recovery values of tetryl from binary mixtures containing both tetryl and ionic species ranged from 95.7% to 110.7%. It was expected that Fe(II) ions, in the presence of H_2_O_2_, would positively affect the proposed method by producing ^•^OH as required by the classical Fenton reaction. However, Fe(II) ions did not interfere with the system, attributable to the limited production of ^•^OH, as the working pH of the proposed method (i.e., pH 5.5) is outside the ideal pH range for the effectiveness of the conventional Fenton reaction (pH 2.0–4.0). These findings are quite consistent with the results obtained in experiments conducted using Fe_3_O_4_NPs containing both Fe(II) and Fe(III) in the search of the optimal catalyst for the proposed method. Acetylsalicylic acid (aspirin), aspartame, household detergent, ᴅ-(+)-glucose, and paracetamol, which have similar appearance to explosives and are used as camouflage during their transport, were investigated for their interference effects on tetryl at concentrations of 1- and 10-fold by mass. According to the data in Table S2, the recovery values for tetryl in binary mixtures with camouflage materials ranged between 90.6 and 107.4%. The fact that these potential interferents have no remarkable effect on the proposed method demonstrates that this assay is quite robust and can be accurately applied to complex matrices.

#### Recovery of tetryl and NTO from soil sample

The extraction of tetryl and NTO from soil samples contaminated with tetryl and NTO, and from munition samples mixed with TNT (i.e., Tetritol and TNTO), was successfully performed (Table S3). Using the proposed method, tetryl was recovered at 101.2% and 102.7% from tetryl- and Tetritol-contaminated soil samples and NTO was recovered at 98.6% and 97.4% from NTO- and TNTO-contaminated soil samples, respectively.

#### Practical smartphone application of the recommended method

**Fig. 6:**
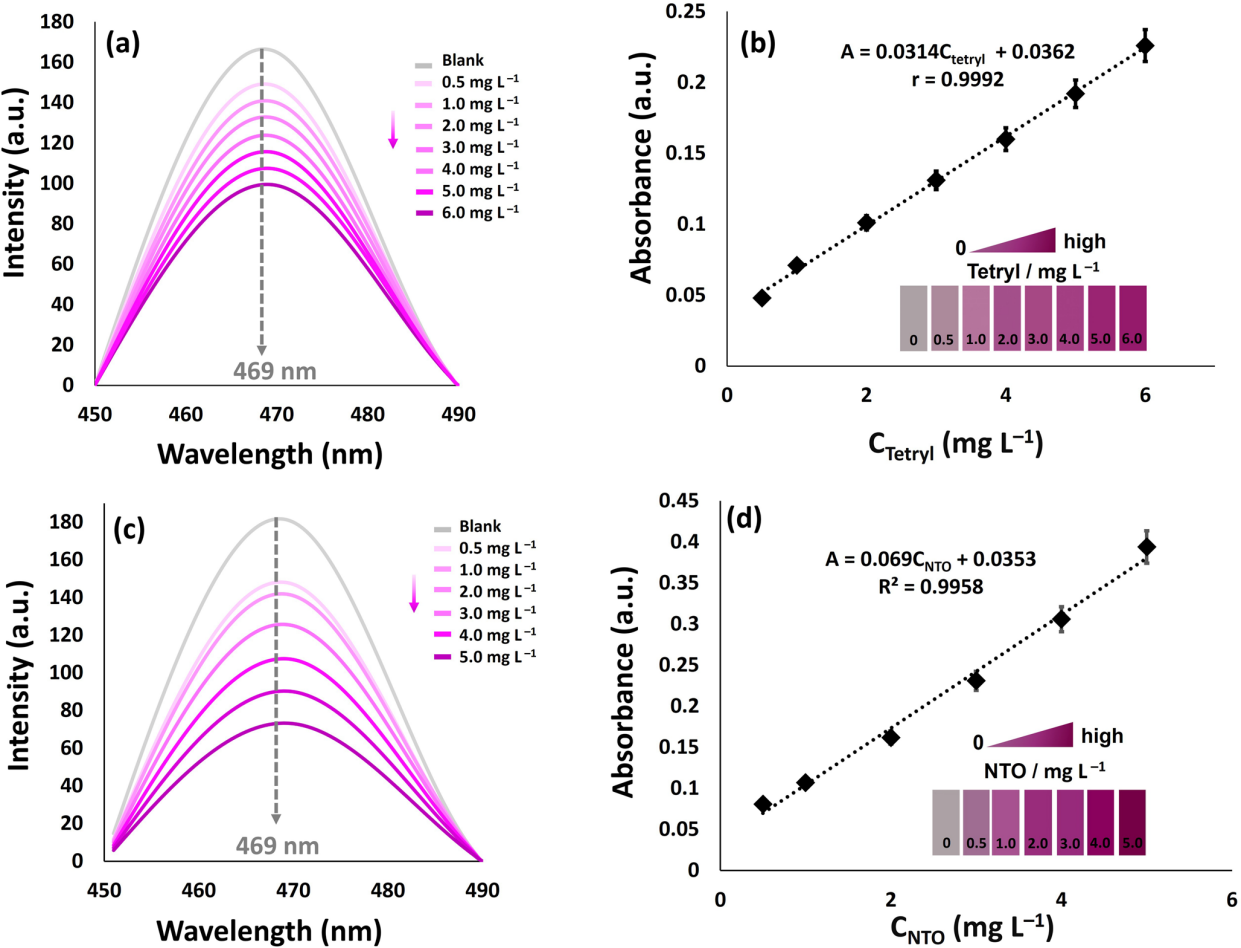
(**a**) Smartphone color intensity spectra obtained by applying the Griess reagent after the decomposition of tetryl (0.50–6.0 mg L^─1^, final conc.) solutions by the proposed Fenton-like oxidation, (**b**) plot of the absorbance at λ_max_ = 469 nm as a function of the final concentration of tetryl (0.50–6.0 mg L^─1^, final conc.), (**c**) smartphone color intensity spectra obtained by applying the Griess reagent after the decomposition of NTO (0.50–5.0 mg L^─1^, final conc.) solutions by proposed Fenton-like oxidation, (**d**) plot of the absorbance at λ_max_ = 469 nm as a function of the final concentration of NTO (0.50–5.0 mg L^─1^, final conc.)

For the smartphone application, the proposed method was applied to tetryl and NTO solutions, with measurements taken using the “colorimeter” app on a Samsung Galaxy A54 smartphone running Android. As shown in Figs. 6a&c, a notable decrease in color intensity at 469 nm occurs as the amount of pink-colored azo dye increases with increasing tetryl and NTO concentrations. To evaluate the results more effectively, the color intensity values at 469 nm were converted to absorbance using the equation (Eq. 3). These absorbance values were plotted against increasing tetryl and NTO concentrations at 469 nm, as shown in Figs. 6b&d, a good linear relationship was observed within the 0.5–6.0 mg L^–1^ tetryl and 0.5–5.0 mg L^–1^ NTO concentration ranges.

3 $${A}=-{log}\raisebox{1ex}{$\:{I}$}\!\left/\:\!\raisebox{-1ex}{${{I}}_{0}$}\right.$$

where A represents absorbance, while I and I_0_ correspond to the intensities of the sample and blank solutions, respectively.

The analytical performance parameters of smartphone applications are detailed in Table S4 of the Supplementary Information.

## Statistical comparison of the proposed method against the reference LC − MS/MS methods

The proposed method procedures were validated against the LC–MS/MS methods described in the Supplementary Information [35, 36]. To evaluate the accuracy and precision of the proposed method, the mean and variance values of the two populations were calculated using data obtained from applying the proposed method and the reference LC-MS/MS method to five different commercial soil samples (sandy soil) contaminated with tetryl and NTO, respectively, under the same conditions; then, Student’s t- and F-tests were performed by applying a series of mathematical calculations including sample calculations footnoted in Table S5. Comparison of the test results for tetryl- and NTO-contaminated soil samples showed no significant difference at 95% confidence level for Student’s t- and F-tests, respectively.

## Comparison of the developed method with other colorimetric methods for tetryl and NTO detection

Colorimetric methods for the determination of tetryl and NTO are available in the literature, and their detection mechanisms and analytical performance parameters are summarized in Table S6. The principle of tetryl determination in colorimetric studies is based on the formation of a Meisenheimer-type charge transfer complex, and both TNT and tetryl have been determined using these colorimetric approaches (Table S6). Compared to colorimetric methods that yielded positive analytical signals for both TNT and tetryl, the proposed method is notable in the literature for its unique mechanism of detecting tetryl, particularly being the first study to specifically and selectively detect tetryl in formulations where both compounds are present. As the other explosives were shown not to interfere, the developed method stands out as a simple, low-cost approach that offers selective and sensitive detection of the target analytes, tetryl and NTO, which are separable by solubility differences prior to Griess colorimetric measurement.

## Conclusion

In the developed method, single-step‒synthesized Cu_2_O@CuONC-assisted Fenton-like decomposition followed by nitrite-mediated colorimetry provides a sensitive and selective colorimetric determination of tetryl and NTO. The developed method is noteworthy for offering several innovations: (i) being the first method to use mixed-valent CuO_2_@CuONC with superior catalytic performance that displays self-reduction (with oxygen evolution) in alkaline medium without any external reducing agents, (ii) providing a unique mechanism for detecting tetryl and NTO simultaneously, (iii) being an innovative alternative to the traditional mechanism of Meisenheimer complex formation for tetryl determination interfered by other nitro-aromatics, and (iv) being a robust method unaffected by most environmental conditions and contaminants. If the degradation of tetryl were to be carried out in alkaline medium, where the N-NO_2_ bond scission mechanism would be diverted from essentially free radical-driven scission (typical of neutral/acidic AOPs: Advanced Oxidation Processes through a Cu-driven Fenton reaction) toward nucleophilic base (OH^−^)‒induced elimination, then other nitramines like RDX and HMX would cleave and liberate nitrite, which would adversely affect the selectivity of the current assay. So, the proposed Cu-driven Fenton degradation (at pH 5.5) of tetryl *via* N-NO_2_ bond cleavage to release nitrite quantifiable by Griess colorimetry is the first of its kind in indirect determination of tetryl. A set of characterization studies (STEM, SEM-EDX, XPS, UV-Vis, ATR-FTIR) was conducted to clarify the size and morphology and to confirm the presence of Cu_2_O@CuONC. The catalytic performance of Cu_2_O@CuONC was compared to that of Fe_3_O_4_NPs, and the results showed that Cu_2_O@CuONC exhibit superior catalytic activity for the proposed method. Studies on elucidation of reactive species have shown that ^•^OH as the leading reactive species of the system directly affects nitrite formation *via* tetryl and NTO degradation, and actively serves the system’s mechanism. The proposed approach demonstrated good selectivity toward other energetic compounds and was successfully applied to munition samples and contaminated soil. This study has the potential to make a significant contribution to environmental monitoring of explosive residues, to address critical environmental and public safety concerns, and to inspire innovative studies that enable the simultaneous determination of sensitive and insensitive energetic materials with high sensitivity and selectivity.

## Supplementary Information

Below is the link to the electronic supplementary material.

Supplementary Material 1 (DOCX 981 KB)

## Data Availability

No datasets were generated or analysed during the current study.
