# Supplementary material for: Fenton-like oxidation–driven dual-mode detection of 2,4,6- trinitrophenyl-N-methylnitramine (tetryl) and 3-nitro-1,2,4-triazole-5-one (NTO) using single-step synthesized Cu2O@CuO nanocomposite
Source: Mikrochim Acta. 2026 Jun 12;193(7):467. doi: 10.1007/s00604-026-08203-3 (PMC13263217; doi:10.1007/s00604-026-08203-3)
Supplement: Supplementary file 1 — Supplementary Material 1 (DOCX 981 KB) [file 604_2026_8203_MOESM1_ESM.docx]

**ELECTRONIC SUPPLEMENTARY INFORMATION**

**Fenton-like oxidation–driven dual-mode detection of 2,4,6- trinitrophenyl-N-methylnitramine (tetryl) and 3-nitro-1,2,4-triazole-5-one (NTO) using single-step synthesized Cu₂O@CuO nanocomposite**

Kader Can^a,b^, Ayşem Üzer^a,^*, Reşat Apak^a,c,^**

^a^Department of Chemistry, Faculty of Engineering, Istanbul University-Cerrahpaşa, 34320 Avcilar, Istanbul, Türkiye.

^b^İstinye University, Faculty of Pharmacy, Department of Analytical Chemistry, 34010 İstanbul, Türkiye

^c^Turkish Academy of Sciences (TUBA), Bayraktar Neighborhood, Vedat Dalokay St. No:112, Çankaya, 06690 Ankara, Türkiye.

*Corresponding author: Ayşem Üzer, E-mail: [auzer@iuc.edu.tr](about:blank)

**Co-corresponding author: Reşat Apak, E-mail: [rapak@istanbul.edu.tr](about:blank); Tel.: +90-212-4737028

Table of Contents

[Abbreviations S2](#_Toc231228777)

[Materials and Chemicals S2](#_Toc231228778)

[Instrumentations S2](#_Toc231228779)

[Preparation of Solutions S3](#_Toc231228780)

[Synthesis of Fe_3_O_4_NPs S3](#_Toc231228781)

[Elucidation of the Sensing Mechanism via Reactive Oxygen Species-Based Tests S4](#_Toc231228782)

[Hypothetical Reaction Mechanism for -N(CH_3_)-NO_2_ Bond Cleavage to Produce Nitrite S4](#_Toc231228783)

[Selectivity and Interference Studies S5](#_Toc231228784)

[Extraction-Based Recovery of Tetryl from the Tetryl–NTO Binary Synthetic Mixture S5](#_Toc231228785)

[Reference LC−MS/MS Conditions S6](#_Toc231228786)

[Supplementary Figures S7](#_Toc231228787)

[Supplementary Tables S15](#_Toc231228788)

[Supplementary References S21](#_Toc231228789)

Abbreviations

Cu_2_O/CuO nanocomposite (Cu_2_O/CuONC), magnetite nanoparticles (Fe_3_O_4_NPs) sulfanilamide (SA), N-(1-Naphthyl)ethylenediamine (NED) terephthalic acid (TA), nitrobule tetrazolium (NBT), acetic acid–sodium acetate (HAc/NaAc), ethanol (EtOH), hydroxyl radicals (^•^OHs), superoxide anion radicals (O_2_^•–^), 2,4,6-trinitrophenyl-N-methylnitramine (tetryl), 3-nitro-1,2,4-triazole-5-one (NTO), 2,4,6-trinitrotoluene (TNT), 2,4,6-trinitrophenol (TNP), 1,3,5-trinitroperhydro-1,3,5-triazine (RDX), 1,3,5,7-tetranitro-1,3,5,7-tetraazacyclooctane (HMX), pentaerythritol tetranitrate (PETN), 4-amino-2,6-dinitrotoluene (4A-DNT), 1,3,5-trinitrobenzen (TNB), nitroguanidine (NQ), ammonium nitrate (NH_4_NO_3_), liquid chromatography – tandem mass spectrometry (LC–MS/MS)

Materials and Chemicals

The energetic materials used all over the study, TNT, tetryl, 2,4,6-trinitrophenol (TNP), RDX, 1,3,5,7-tetranitro-1,3,5,7-tetraazacyclooctane (HMX), pentaerythritol tetranitrate (PETN), 1,3,5-trinitrobenzene (TNB) and 4-amino-2,6-dinitrotoluene (4A-DNT) were graciously supplied by the Mechanical and Chemical Industry Corporation (Makine Kimya Endüstrisi Kurumu-MKEK; Ankara, Turkey) through previous projects. NTO was kindly supplied by Sabancı University (Istanbul, Turkey) as an originally synthesized product from the Chemistry Department's laboratories. Nitroguanidine (NQ) was obtained from Sigma-Aldrich (St. Louis, Missouri, USA) and ammonium nitrate (NH_4_NO_3_) from Merck (Darmstadt, Germany).

All chemical substances used in the study are of analytical grade and include: copper(II) acetate, glacial acetic acid, sodium hydroxide (NaOH), 30% hydrogen peroxide (H_2_O_2_), sulfanilamide (SA), sodium acetate (CH_3_COONa), and phosphoric acid (H_3_PO_4_). All these chemicals were purchased from Merck (Darmstadt, Germany). Additionally, N-(1-naphthyl)ethylenediamine (NED) was purchased from Aldrich (St. Louis, Missouri, USA).

For real sample analyses, clean sandy soil (CLN SOIL-1), used as certified reference material, was provided by RTC (Laramie, WY, USA).

Instrumentations

All spectrophotometric measurements and optical characterizations were performed using a Shimadzu UV-1800 ultraviolet-visible spectrophotometer (Shimadzu, Japan) with 10 mm path length Hellma Suprasil quartz cuvettes (Hellma Analytics, USA). Scanning transmission electron microscopy (STEM) and scanning electron microscopy-energy dispersive X-ray spectroscopy (SEM-EDX) measurements were performed on an FEI Quanta FEG 450 (Hillsboro, Oregon, USA). X-ray photoelectron spectroscopy (XPS) analyses were performed using a Thermo Fisher K-Alpha spectrometer (Thermo Fisher, USA) equipped with a monochromatic Al Kα X-ray source (hν = 1486.6 eV). Fourier-transform infrared (FTIR) spectra were collected on a Shimadzu IRTracer-100 FTIR Spectrometer combined with Attenuated Total Reflectance (ATR) accessory. The necessary incubation steps during the degradation process of blank and/or real samples were performed using a temperature-controlled Wisd WiseBath water bath. Method validation studies were performed using a liquid chromatography-tandem mass spectrometry (LC–MS/MS) system that consisted of a Shimadzu LC–20A liquid chromatograph and a Shimadzu LC-MS–8040 mass spectrometer.

Preparation of Solutions

Solutions prepared for the synthesis of Cu_2_O/CuO nanocomposite: Copper (II) acetate (Cu(CH_3_COO)_2_), used as a copper source, was prepared with ultrapure water at a concentration of 0.02 mol L^─1^. NaOH, which plays an active role in the synthesis of Cu_2_O/CuONC, was prepared with ultrapure water at a concentration of 0.10 mol L^─1^.

Preparation of Griess reagent: Griess reagent was prepared following the method suggested by Sawicki and Scaringelli [1] . Accordingly, a 1.0% (w/v) SA solution was prepared in 5% (v/v) phosphoric acid solution. A 0.1% (w/v) NED solution was prepared in ultrapure water. Finally, these two solutions were mixed in equal volumes (1:1, v/v) to obtain the Griess reagent.

All stock solutions of energetic compounds were prepared in ethanol at a concentration of 2000 mg L^─1^ active substance. Working solutions of 50.0 mg L ^─1^ used for tetryl and NTO were prepared by diluting appropriate volumes of the relevant stock solutions with pure water.

Synthesis of Fe_3_O_4_NPs

The Fe_3_O_4_NPs were prepared via the coprecipitation synthesis procedure reported in the literature [2]. First, 50 mL of 1 M ferric chloride (FeCl_3_) aqueous solution and 10 mL of 2 M ferrous chloride (FeCl_2_) aqueous solution in 2 M HCl were mixed and deoxygenated by purging with nitrogen gas for at least 10 min. Second, the mixed solution of ferrous and ferric salts was added dropwise into 500 mL of 0.7 M oxygen-free aqueous ammonia solution under vigorous stirring for 30 min at room temperature under a nitrogen atmosphere. The formed black Fe_3_O_4_ colloidal particles were separated by filtration and further washed three times with water.

Elucidation of the Sensing Mechanism via Reactive Oxygen Species-Based Tests

**Terephthalic Acid Test.** In the proposed method, the TA test was performed in the presence and absence of 25 mg L^–1^ tetryl to determine the presence of hydroxyl radicals (^•^OH) and their contribution to the mechanism. For this purpose, 50 µL of 1000 mg L^–1^ Cu_2_O@CuONC solution was transferred to a test tube, then 50 µL of 1.0 mol L^–1^ H_2_O_2_ and 0.2 mL of 1.0 mol L^–1^ acetic acid-sodium acetate buffer (pH 5.5) were added. Subsequently, 0.15 mL of 20 mmol L⁻¹ TA (prepared in 0.10 mol L⁻¹ NaOH) and 0.20 mL of 25 mg L^–1^ tetryl solution (for the sample solution) and/or 0.2 mL of ethanol (for the reference solution) were added, and the final volume was adjusted to 1.50 mL with ethanol (EtOH). The solution was incubated at 60 °C in a water bath for 40 minutes. After incubation, the fluorescence spectra of the reference and sample solutions were measured at an excitation wavelength of 315 nm under room conditions.

**Nitroblue Tetrazolium (NBT)Test.** In the proposed method, the NBT test was performed in the presence and absence of 25 mg L^–1^ tetryl to determine the presence of superoxide anion radicals (O_2_^•–^) and their contribution to the mechanism. In this regard, 50 µL of 1000 mg L^–1^ Cu_2_O@CuONC solution was taken and placed in a test tube, followed by the addition of 50 µL of 1.0 mol L^–1^ H_2_O_2_ and 0.2 mL of 1.0 mol L^–1^ acetic acid–sodium acetate buffer (pH 5.5). Afterwards, 0.30 mL of 300 µmol L^–1^ NBT and 0.20 mL of 25 mg L^–1^ tetryl solution (for the sample solution) and/or 0.2 mL of ethanol (for the reference solution) were added, and the final volume was adjusted to 1.50 mL with EtOH. The solution was then incubated in a 60 °C water bath for 40 minutes. After incubation, the absorbance measurements of the reference and sample solutions were made against water using a UV-visible spectrophotometer.

Hypothetical Reaction Mechanism for -N(CH_3_)-NO_2_ Bond Cleavage to Produce Nitrite

The Cu(I)-driven Fenton reaction generates the strong oxidant hydroxy radicals (•OH), which would attack the N-NO_2_ bond possessing a lower bond dissociation energy and a higher susceptibility to radical attacks compared to aromatic C-NO_2_ bonds. This is a multi-step reaction mechanism consisting of several hypothetical stages [3] outlined below:

(i) Radical generation and initial •OH attack: In the presence of H_2_O, •OH (generated through a Cu-driven Fenton reaction) can add to the amine nitrogen of tetryl as the electron-rich site or attack the CH_3_-group, forming a reactive radical intermediate;

(ii) Electron transfer (ET) and nitro-nitrite conformational isomerization: the oxidative medium induces a rearrangement through the change in the electron density of nitramine. In this step, tetryl is expected to transfer a single electron (SET) to the oxidant, producing a transient species (*i.e.* cationic tetryl radical) and giving way to nitro-nitrite isomerization, converting from the more stable (N-NO_2_) into the less stable conformer (-N-O-NO);

(iii) Homolytic (N-O) (or N-N) bond breakage, releasing nitrogen dioxide radicals (•NO_2_) into solution;

(iv) Radical quenching and reduction to nitrite (NO_2_^-^): this step involves the conversion of •NO_2_ radicals to stable nitrite ions through secondary reactions, such as electron capture from matrix and disproportionation-hydrolysis:

(NO_2_ + NO **→** N_2_O_3_ and N_2_O_3_ + H_2_O → 2 H^+^ + 2NO_2_^-^)

Selectivity and Interference Studies

**Selectivity Study.** In order to investigate the selectivity of the proposed method, the potential interference effects of different types of explosives found in soil and/or seized ammunition samples were evaluated. For this purpose, the proposed method was applied to selected energetic materials, including TNT (also at a 50-fold ratio), RDX, HMX, TNP, PETN, TNB, 4A-DNT, NTO (as the other analyte), NQ, and NH_4_NO_3_, at 1- and 10-fold mass ratios in the presence (2.0 mg L^–1^, final conc.) and absence of tetryl. Afterwards, the selectivity of the method was evaluated and the recovery values (%) of tetryl were calculated using the absorbance values at a wavelength of 542 nm

**Interference Study.** In order to evaluate the applicability of the proposed method in complex environmental systems, the potential interference effects of cationic {Na^+^, K^+^, Ca^2+^, Mg^2+^, Al^3+^, Fe (II) and Fe (III)}and anionic (Cl^–^, NO_3_^–^, SO_4_^2–^ and CO_3_^2–^) species present in soil and water samples, as well as the potential interference effects of camouflage materials (acetylsalicylic acid (aspirin), paracetamol, sugar (*e.g.,* ᴅ-(+)-glucose) and household powder detergents), carried by passengers as personal items in their hand luggage, were investigated. For this purpose, the proposed method was applied to certain water and soil ions at 1-, 10- and 100-fold mass ratios (for Al^3+^ and CO_3_^2–^, 1-, 10-, and 50-fold) of tetryl and possible camouflage materials at varying molar ratios (e.g., 1-, 10-, 100-fold of tetryl). Afterwards, the selectivity of the method was evaluated and the recovery values (%) of tetryl were calculated using the absorbance values at a wavelength of 542 nm.

Extraction-Based Recovery of Tetryl from the Tetryl–NTO Binary Synthetic Mixture

To effectively separate NTO from the tetryl–NTO binary synthetic mixture, a solubility difference-based separation technique was proposed, utilizing the water solubility of tetryl and NTO (tetryl solubility in water is 0.008 g L^−1^ / 25 °C; NTO solubility in water is 16.6 g L^−1^ / 25 °C) [4, 5]. For this approach, 0.01 g of synthetically mixed tetryl and NTO was initially treated with 5 mL of ultrapure water and stirred for 5 minutes in a shaker. The resulting mixture was then centrifuged at 5000 rpm for 5 minutes. After centrifugation, the supernatant containing NTO was carefully removed. After separating the NTO from the synthetic mixture, the undissolved tetryl remaining in the aqueous phase was dissolved in ethanol and its final volume was brought to 10 mL. Finally, the tetryl solution was diluted to 50.0 mg L^–1^ and the proposed method was applied. The recovery obtained from the extraction of tetryl from the tetryl–NTO binary mixture is given in Table S1.

Reference LC−MS/MS Conditions

The validation of the colorimetric method developed for the determination of tetryl and NTO was evaluated comparatively with methods reported in the literatüre [6, 7].

**Tetryl Determination*.*** 500.0 mg L^−1^ of tetryl stock solution was prepared in ethanol and then diluted within the concentration range of working solutions, *i.e.,* 10 – 100 µg L^−1^, with ethanol. LC was equipped with a Restek Ultra-AQ C-18 reverse phase column (100 × 2.1 mm ID, 3 μm particle size). Analyses were performed using a flow rate of 0.30 mL min^–1^ and an injection volume of 15.0 µL. The column was eluted under isocratic elution conditions of 5% mobile phase A of 5.0 mmol L^–1^ ammonium acetate (NH_4_Ac) in water and 95% mobile phase B of 5.0 mmol L^–1^ NH_4_Ac in methanol. The autosampler temperature was kept at 15 °C, the column was kept at 40 °C. LC−MS/MS analysis was performed using the negative ion mode electrospray ionization method. The product ion and precursor ion were 219.1 m/z and 121.2 m/z for tetryl, respectively (collision energy: 10.0 eV) [6].

**NTO determination.** 500.0 mg L^–1^ NTO stock solution was prepared in ethanol and diluted within the concentration range of 10.0 – 200.0 µg L^–1^ using methanol. An LC–MS/MS system equipped with a Restek Ultra AQ C18 (3 mm × 100 mm × 2.1 mm ID) reversed-phase column was used. At a flow rate of 0.30 mL min^–1^, the column was eluted with the following gradient: from 0 to 5 minutes, mobile phase A (2.5 mmol L^–1^ NH_4_Ac in water) decreased linearly from 95% to 5%, while mobile phase B (2.5 mmol L^–1^ NH_4_Ac in methanol) increased linearly from 5% to 95%. From 5 to 8 minutes, the mobile phase composition was maintained at 5% A and 95% B for 3 minutes. The injection volume was 15 µL, and the column oven temperature was set to 40 °C. Statistical comparisons between the results of the recommended (nanospectrophotometric) and reference (LC–MS/MS) methods were performed using Student’s t- and F-tests [7].

Supplementary Figures


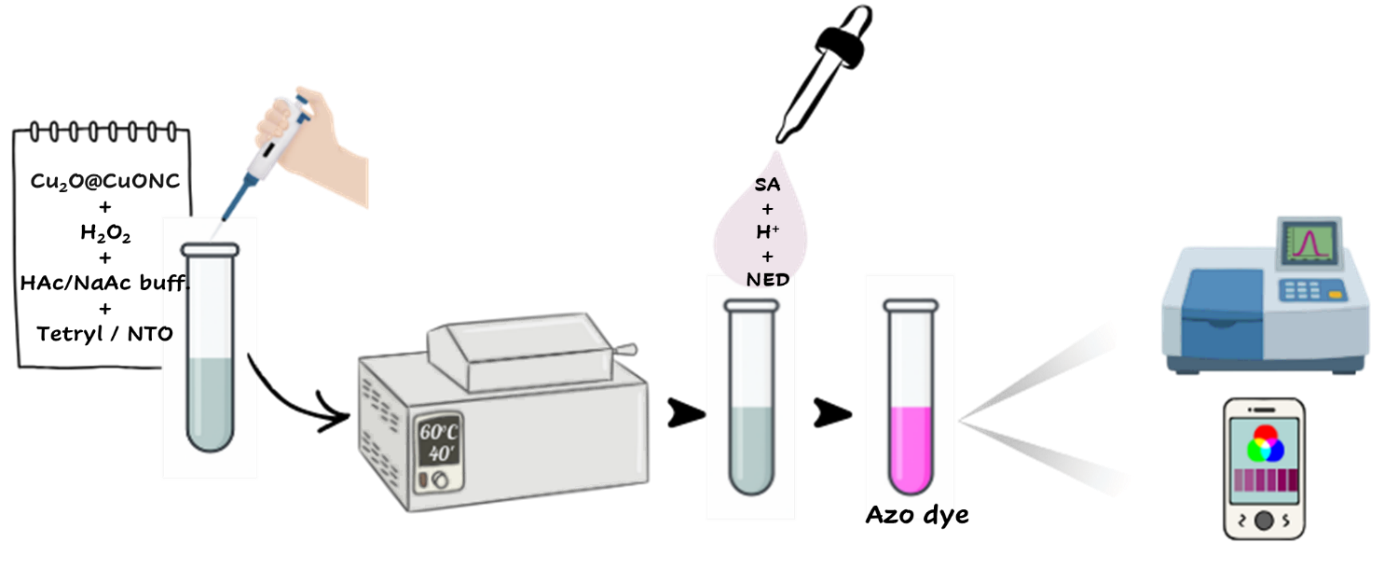


**Scheme S1.** Schematic illustration of applying the recommended procedure to tetryl and/or NTO determination.

**
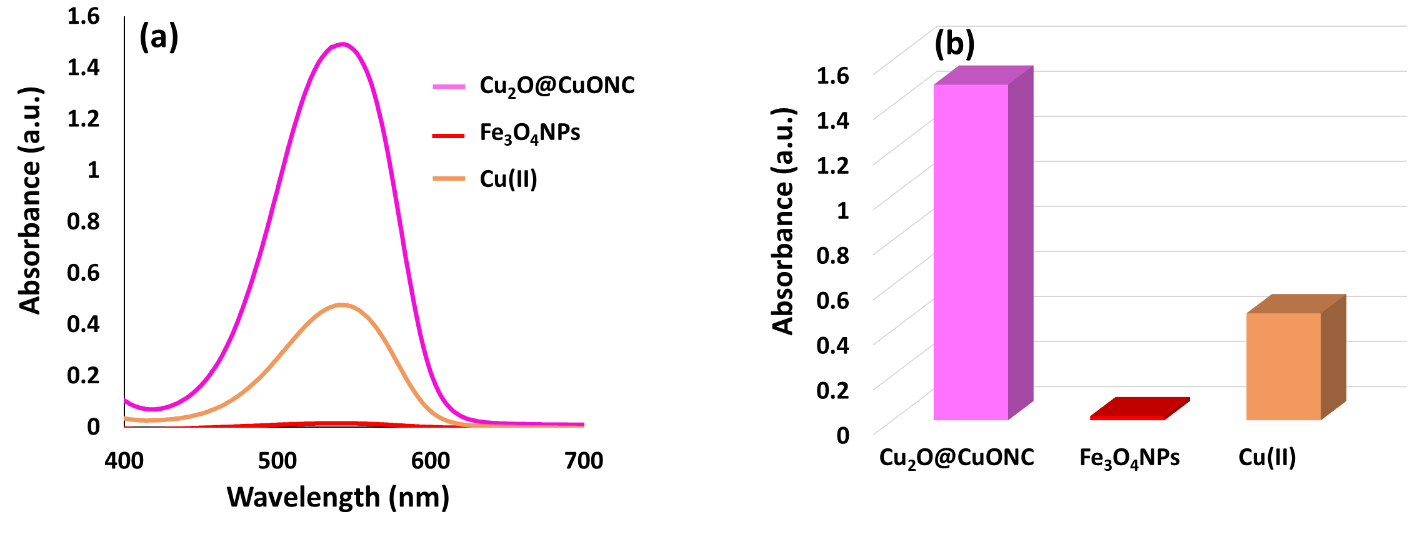
**

**Fig. S1** a) UV-visible absorption spectra and (b) bar diagrams obtained by applying the method to 10.0 mg L^─1^ tetryl solution for comparison of the catalytic activity of Cu_2_O@CuONC, Fe_3_O_4_NPs and Cu(II) {Exp. conditions: [H_2_O_2_]_initial_ = 0.5 mol L^─1^, [Tetryl]_final_ = 10.0 mg L^─1^, pH 5.5 HAc/NaAc buffer, incubation temperature and time: 60 ^o^C and 20-min, V_Griess reagent_ = 2.0 mL}.

**
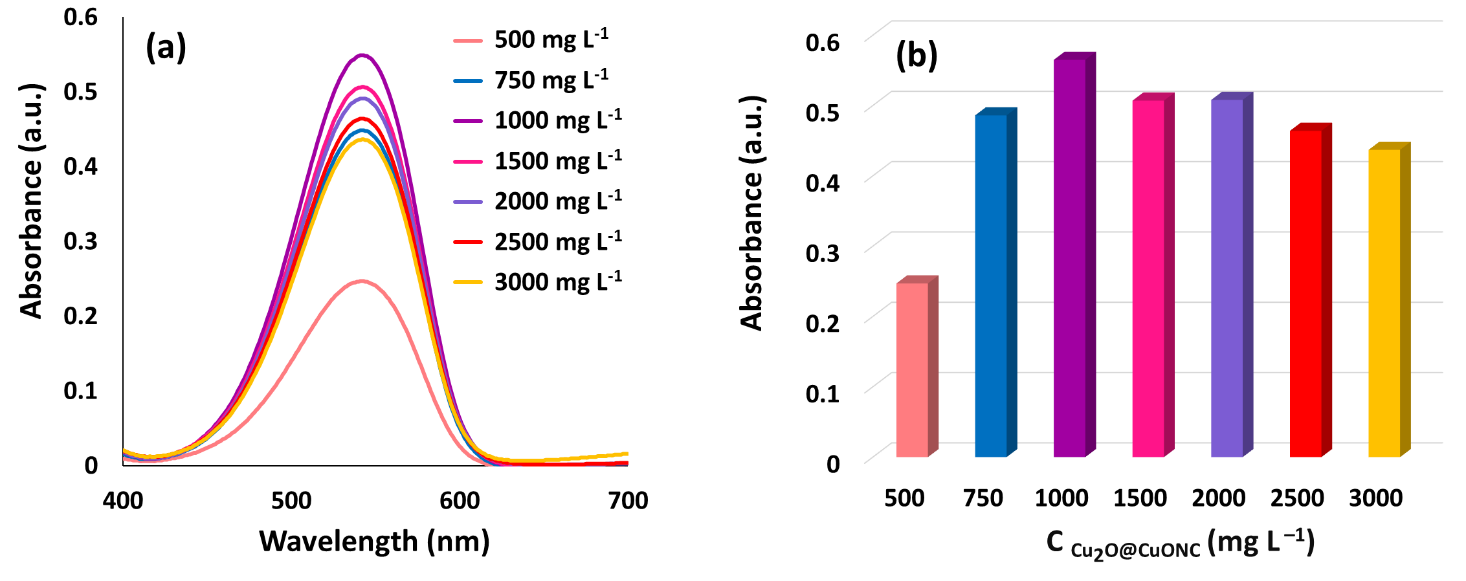
**

**Fig. S2** (a) UV-visible absorption spectra and (b) bar diagrams obtained by applying the method to a 3.5 mg L^─1^ tetryl solution at different Cu_2_O@CuONC concentrations (500 – 3000 mg L^─1^) {Exp. conditions: [H_2_O_2_]_initial_ = 0.5 mol L^─1^, [Tetryl]_final_ = 3.5 mg L^─1^, pH 5.5 HAc/NaAc buffer, incubation temperature and time: 60 ^o^C and 30-min, V_Griess reagent_ = 2.0 mL}.

**
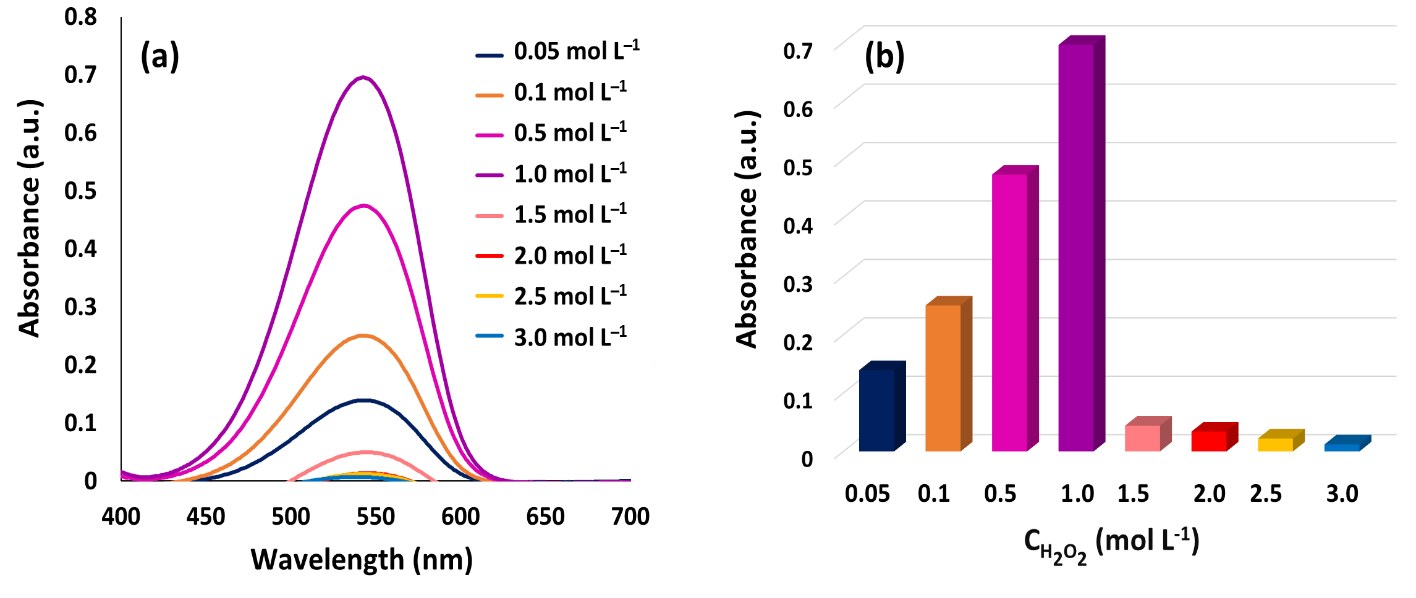
**

**Fig. S3** (a) UV-visible absorption spectra and (b) bar diagrams obtained by applying the method to 3.5 mg L^─1^ tetryl solution at different H_2_O_2_ concentrations (0.05 – 3.0 mol L^─1^) {Exp. conditions: [Cu_2_O@CuONC]_initial_ = 1000 mg L^─1^, [Tetryl]_final_ = 3.5 mg L^─1^, pH 5.5 HAc/NaAc buffer, incubation temperature and time: 60 ^o^C and 30-min, V_Griess reagent_ = 2.0 mL}.

**
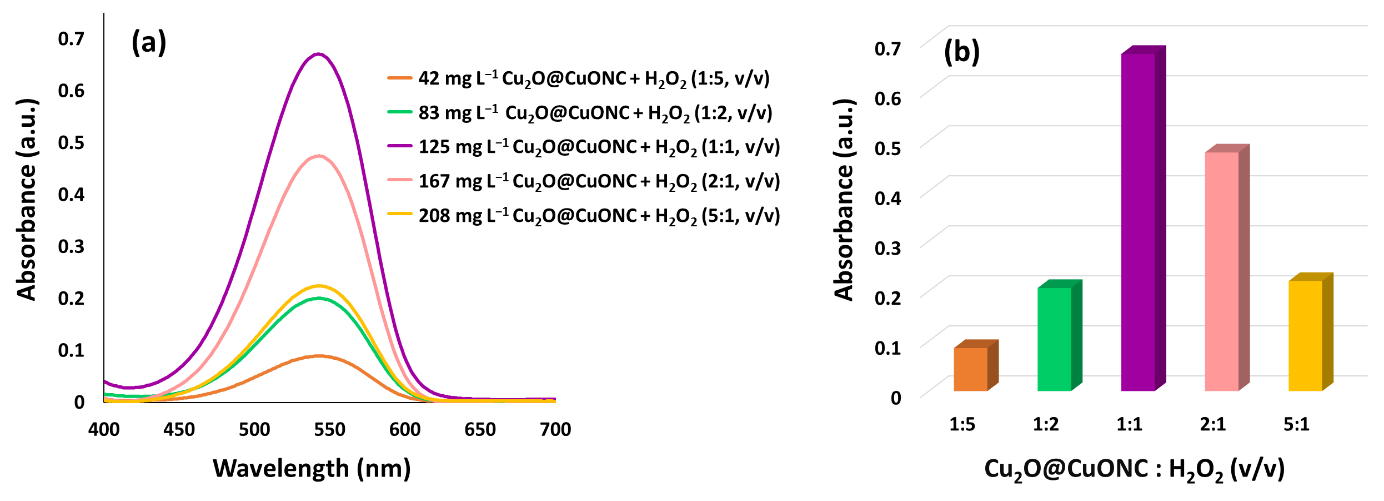
**

**Fig. S4 (a)** UV-visible absorption spectra and (b) bar diagrams obtained by applying the method to a 3.5 mg L^─1^ Tetryl solution of Cu_2_O@CuONC (with the corresponding final concentrations of Cu_2_O@CuONC indicated for each volume ratio) and H_2_O_2_ at different volume ratios {Exp. conditions: [Cu_2_O@CuONC]_initial_ = 1000 mg L^─1^, [H_2_O_2_]_initial_ = 1.0 mol L^─1^, [Tetryl]_final_ = 3.5 mg L^─1^, pH 5.5 HAc/NaAc buffer, incubation temperature and time: 60 ^o^C and 30-min, V_Griess reagent_ = 2.0 mL}.

**
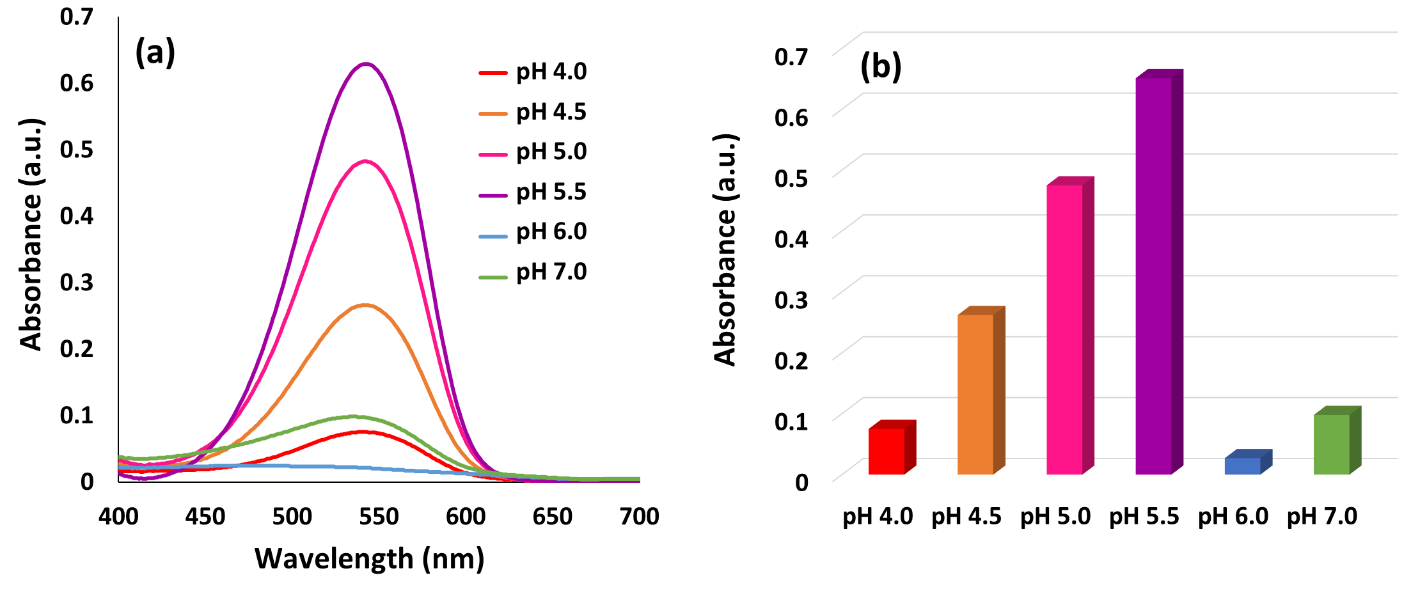
**

**Fig. S5** (a) UV-visible absorption spectra and (b) bar diagrams obtained by applying the method to 3.5 mg L^─1^ tetryl solution at different pH {Exp. conditions: [Cu_2_O@CuONC]_initial_ = 1000 mg L^─1^, [H_2_O_2_]_initial_ = 1.0 mol L^─1^, [Tetryl]_final_ = 3.5 mg L^─1^, incubation temperature and time: 60 ^o^C and 30-min, V_Griess reagent_ = 2.0 mL}.


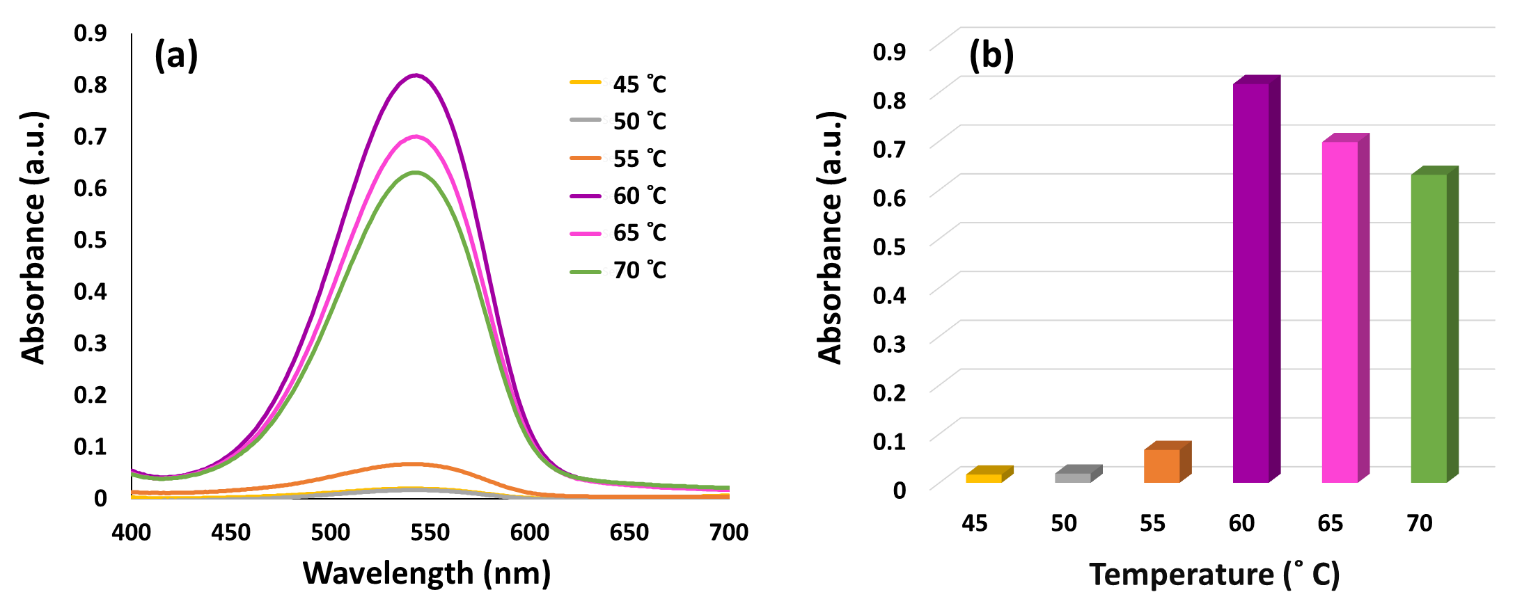


**Fig. S6** (a) UV-visible absorption spectra and (b) bar diagrams obtained by applying the method to 3.5 mg L^─1^ tetryl solution at different temperatures (45.0 – 70.0 ^o^C) {Exp. conditions: [Cu_2_O@CuONC]_initial_ = 1000 mg L^─1^, [H_2_O_2_]_initial_ = 1.0 mol L^─1^, [Tetryl]_final_ = 3.5 mg L^─1^, pH 5.5 HAc/NaAc buffer, incubation time: 30-min, V_Griess reagent_ = 2.0 mL}.

**
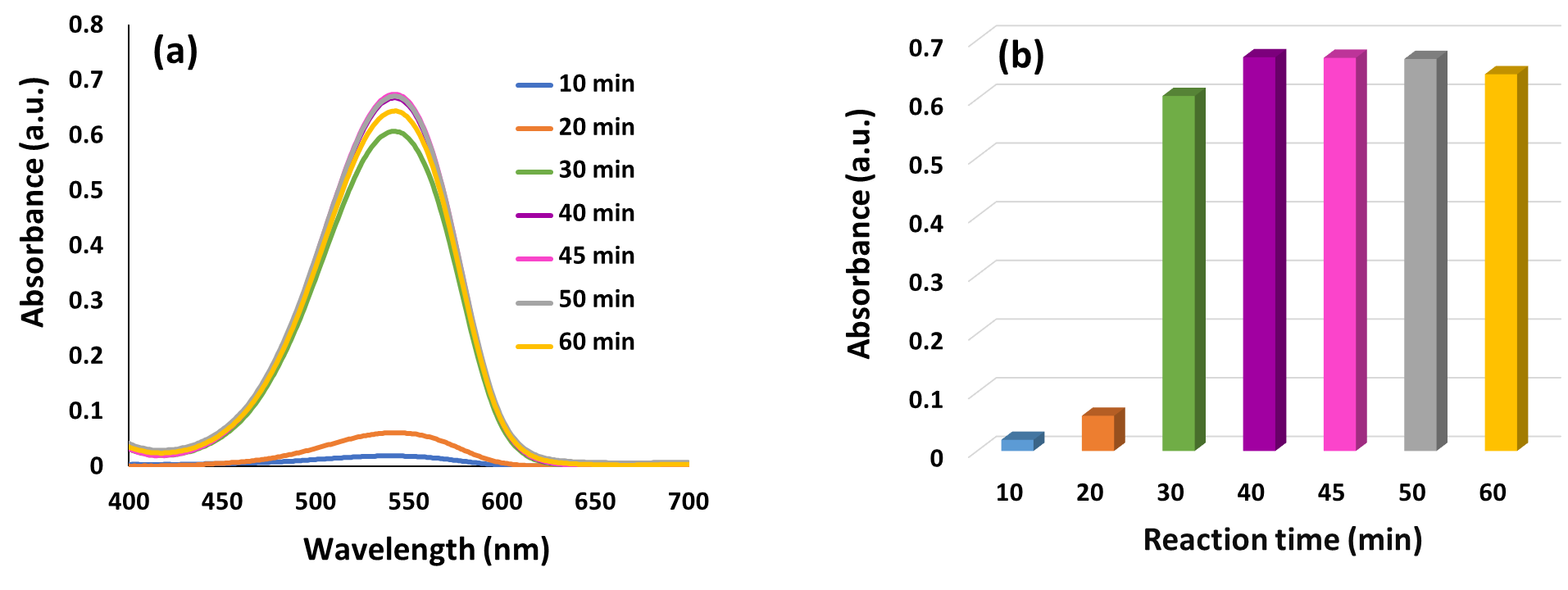
**

**Fig. S7** (a) UV-visible absorption spectra and (b) bar diagrams obtained by applying the method to 3.5 mg L^─1^ tetryl solution at different reaction times {Exp. conditions: [Cu_2_O@CuONC]_initial_ = 1000 mg L^─1^, [H_2_O_2_]_initial_ = 1.0 mol L^─1^, [Tetryl]_final_ = 3.5 mg L^─1^, pH 5.5 HAc/NaAc buffer, incubation temperature: 60 ^o^C, V_Griess reagent_ = 2.0 mL}.

Supplementary Tables

**Table S1.** Recovery values (%) obtained by applying the developed method to different types and mass ratios of explosive mixtures containing tetryl (at 2.5 mg L^−1^, final conc.).

| **Synthetic mixture** | **Mass ratio**  **(Tetryl/other explosives)** | **A_542nm_** | **Recovery ± SD (%)** |
| --- | --- | --- | --- |
| **Tetryl** | – | 0.507 | – |
| **Tetryl: NTO** | 1:1 | 1.134 | 223.6 ± 1.7 |
|  | 1:1 | 0.514 | **101.4 ± 1.1**^a^ |
| **Tetryl: TNT** | 1:1 | 0.518 | 102.2 ± 0.9 |
|  | 1:10 | 0.528 | 104.1 ± 1.3 |
|  | 1:50 | 0.541 | 106.7 ± 1.7 |
| **Tetryl: TNP** | 1:1 | 0.525 | 103.6 ± 1.2 |
|  | 1:10 | 0.549 | 108.3 ± 0.7 |
| **Tetryl: RDX** | 1:1 | 0.523 | 103.2 ± 1.1 |
|  | 1:10 | 0.517 | 102.0 ± 1.9 |
| **Tetryl: HMX** | 1:1 | 0.520 | 102.6 ± 2.1 |
|  | 1:10 | 0.510 | 100.7 ± 1.4 |
| **Tetryl: PETN** | 1:1 | 0.527 | 104.1 ± 0.8 |
|  | 1:10 | 0.542 | 107.0 ± 0.7 |
| **Tetryl: TNB** | 1:1 | 0.520 | 102.7 ± 0.3 |
|  | 1:10 | 0.524 | 103.4 ± 0.8 |
| **Tetryl: 4- ADNT** | 1:1 | 0.517 | 102.0 ± 0.9 |
|  | 1:10 | 0.524 | 103.5 ± 0.9 |
| **Tetryl: NQ** | 1:1 | 0.519 | 102.4 ± 0.4 |
|  | 1:10 | 0.522 | 103.1 ± 0.7 |
| **Tetryl: NH_4_NO_3_** | 1:1 | 0.518 | 102.2 ± 0.5 |
|  | 1:10 | 0.533 | 105.2 ± 0.9 |

^a^ interference effect of NTO was eliminated by an extraction process exploiting solubility differences.

**Table S2**. Recovery (%) values obtained by applying the developed method to ionic species and certain camouflage materials along with tetryl (at 2.5 mg L^−1^) at different mass ratios.

| **Interferent** | | **Mass ratio** | **A_542nm_** | **Recovery ± SD (%)** |
| --- | --- | --- | --- | --- |
| **Ionic**  **species** | **Tetryl** | – | 0.514 | – |
|  | **Na^+^** | 1:1 | 0.502 | 97.7±0.9 |
|  |  | 1:10 | 0.510 | 99.5± 1.3 |
|  |  | 1:100 | 0.507 | 98.6± 1.1 |
|  | **Ca^2+^** | 1:1 | 0.564 | 109.7± 0.6 |
|  |  | 1:10 | 0.569 | 110.7± 0.9 |
|  |  | 1:100 | 0.518 | 100.0± 0.8 |
|  | **Mg^2+^** | 1:1 | 0.550 | 107.0± 2.2 |
|  |  | 1:10 | 0.559 | 108.7± 1.8 |
|  |  | 1:100 | 0.506 | 98.4± 2.7 |
|  | **Fe (II)** | 1:1 | 0.561 | 109.1± 5.2 |
|  |  | 1:10 | 0.542 | 105.6± 5.1 |
|  |  | 1:100 | 0.527 | 102.5± 4.9 |
|  | **Fe (III)** | 1:1 | 0.539 | 104.8± 3.8 |
|  |  | 1:10 | 0.543 | 105.6± 4.6 |
|  |  | 1:100 | 0.529 | 102.9± 3.2 |
|  | **Al^3+^** | 1:1 | 0.523 | 101.9± 3.7 |
|  |  | 1:10 | 0.518 | 100.7± 2.6 |
|  |  | 1:50 | 0.503 | 97.8± 2.9 |
|  | **Cl^–^** | 1:1 | 0.487 | 95.7± 0.6 |
|  |  | 1:10 | 0.545 | 106.0± 0.8 |
|  |  | 1:100 | 0.510 | 99.2± 0.7 |
|  | **NO_3_^–^** | 1:1 | 0.495 | 96.3± 1.2 |
|  |  | 1:10 | 0.485 | 94.3± 1.1 |
|  |  | 1:100 | 0.556 | 108.1± 0.9 |
|  | **CO_3_^2–^** | 1:1 | 0.512 | 99.6± 2.2 |
|  |  | 1:10 | 0.557 | 108.3± 2.1 |
|  |  | 1:50 | 0.559 | 108.7± 2.8 |
|  | **SO_4_^2–^** | 1:1 | 0.545 | 105.4± 1.1 |
|  |  | 1:10 | 0.523 | 101.7± 1.8 |
|  |  | 1:100 | 0.520 | 101.2± 2.0 |
| **Camouflage material** | **Acetylsalicylic acid (aspirin)** | 1:1 | 0.474 | 107.3 ± 2.3 |
|  |  | 1:10 | 0.464 | 92.5± 3.1 |
|  | **Aspartame** | 1:1 | 0.523 | 90.6± 2.9 |
|  |  | 1:10 | 0.550 | 102.1± 2.7 |
|  | **Detergent** | 1:1 | 0.512 | 107.4± 5.1 |
|  |  | 1:10 | 0.527 | 102.5± 5.9 |
|  | **ᴅ-(+)-glucose** | 1:1 | 0.525 | 102.9± 4.7 |
|  |  | 1:10 | 0.504 | 102.5± 5.2 |
|  | **Paracetamol** | 1:1 | 0.485 | 98.4± 3.8 |
|  |  | 1:10 | 0.478 | 94.7± 2.9 |

**Table S3.** Recovery (%) values of tetryl and NTO from soil samples contaminated with tetryl, NTO and their munition mixtures.

| **Synthetic mixture** | **Percentage Explosive Compositions (in mass)** | **A_found_**  **_(542 nm)_** | **A_expected_**  **_(542 nm)_** | **Recovery (%)** |
| --- | --- | --- | --- | --- |
| Tetryl^a^ | – | 0.521 | 0.515 | 101.2 |
| Tetritol | 70% Tetryl^a^ + 30% TNT | 0.529 |  | 102.7 |
| NTO^b^ | – | 0.501 | 0.508 | 98.6 |
| TNTO | 50% NTO^b^ + 50% TNT | 0.495 |  | 97.4 |

^a^2.5 mg L^–1^ (final conc.), ^b^2.0 mg L^–1^ (final conc.)

**Table S4.** The analytical performance parameters of the smartphone application for the recommended method.

| **Analyte** | **Linear range^a^** | **Calibration equation and correlation coefficient** | **LOD^b^** |
| --- | --- | --- | --- |
| Tetryl | 0.50 – 6.0 | A_469_ = 0.0314 C_tetryl_ + 0.0362  r = 0.9992 | 75.0 |
| NTO | 0.5 – 5.0 | A_469_ = 0.069 C_NTO_ + 0.0353  r = 0.9958 | 18.0 |

^a^In mg L^–1^ units (final conc.), ^b^Limit of detection, in µg L^–1^ units (LOD = 3σ_bl_/m, σ_bl_ denoting the standard deviation of the blank absorbance at 469 nm, and m showing the slope of the calibration line)

**Table S5.** Statistical comparison of the proposed sensing method with the LC–MS/MS reference assay for tetryl and NTO determination.

| **Analyte** | **Method** | **Mean conc.**  **(mg L^-1^)** | **Std. dev.**  **(σ)** | **Pooled std. dev.^a,b^**  **(S)** | **t_exp._^a,b^** | **t_critic._^a,b^** | **F_exp._^a,b^** | **F_critic._^a,b^** |
| --- | --- | --- | --- | --- | --- | --- | --- | --- |
| Tetryl | Proposed method | 2.58 | 0.00316 | 0.0187 | 1.05 | 2.31 | 1.65 | 6.39 |
|  | LC-MS/MS method | 2.61 | 0.00523 |  |  |  |  |  |
| NTO | Proposed method | 2.08 | 0.0269 | 0.0157 | 1.57 | 2.31 | 4.46 | 6.39 |
|  | LC-MS/MS method | 2.04 | 0.0569 |  |  |  |  |  |

^a^S^2^ = ((n_1_ – 1)s_1_^2^ + (n_2_ – 1)s_2_^2^) / (n_1_ + n_2_ – 2) and t = (ā_1_ – ā_2_) / (S (1/n_1_ + 1/n_2_)^1/2^), where  s_1_ and s_2_ are the standard deviations of the two populations with sample sizes of n_1_ and n_2_, and sample means of ā_1_ and ā_2_ respectively (t has (n_1_ + n_2_ – 2) degrees of freedom); here, n_1_ = n_2_ = 5. ^b^Statistical comparison at 95% confidence level on paired data obtained with the proposed and reference methods (P = 0.05)

**Table S6.** Comparison of the detection mechanisms and analytical performance parameters of the proposed method with those of other reported colorimetric methods for tetryl and NTO detection

| **Target analyte(s)** | **Sensor**  **materials** | **Detection mechanism** | **LOD** | **References** |
| --- | --- | --- | --- | --- |
| TNT/tetryl | DCHA/PVC | The formation of blue (λ_max_ = 530 nm) and orange (λ_max_ = 460 nm) color charge-transfer complexes with TNT and tetryl, respectively, by DCHA, which constitutes the active component of the sensor. | TNT: 3.0 mg L^–1^  Tetryl: 3.8 mg L^–1^ | [8] |
| TNT/tetryl | DACH-TGA modified AuNPs | The plasmonic shift occurring in the sensor due to the reduction in the distance between AuNPs as a result of the formation of Meisenheimer-type charge-transfer complexes between the free amine (–NH₂) group of DACH on the surface and the –NO₂ groups of TNT and tetryl. | TNT: 1.76 pmol L^–1^  Tetryl: 1.74 pmol L^–1^ | [9] |
| TNT/tetryl | CTAB-DDTC modified AuNPs | The formation of Meisenheimer-type charge-transfer complexes with DDTC on the surface of AuNPs, which are purple with TNT (λ_max_ = 534 nm) and orange with tetryl (λ_max_ = 458 nm), results in a bathochromic shift in the SPR absorption band of the nanosensor. | TNT: 35.2 µmol L^–1^  Tetryl: 2.78 µmol L^–1^ | [10] |
| TNT/tetryl | EDA modified MNPs | Formation of Meisenheimer-type charge-transfer complexes between the free –NH₂ group of EDA on the surface of MNPs and the –NO₂ groups of TNT and tetryl  (λ_max_ = 512 nm) | TNT: 0.25 mg L^–1^  Tetryl: 0.1 mg L^–1^ | [11] |
|  |  | The inhibition of the blue (λmax = 650 nm) colored diimine charge-transfer complex (ox.TMB-TMB) formed as a result of •OH produced in the presence of EDA-functionalized MNPs and H₂O₂ oxidizing TMB (ox.TMB) in the presence of TNT and tetryl. | TNT: 0.95 mg L^–1^  Tetryl: 1.75 mg L^–1^ |  |
| TNT/tetryl | Thiram modified AuNPs | The formation of charge-transfer complexes between the tertiary amine groups on the surface of AuNPs and the –NO₂ groups of TNT and tetryl causes a plasmonic shift in the sensor absorption spectrum due to AuNPs aggregation. | TNT: 5.0 µg L^–1^  Tetryl: 5.0 µg L^–1^ | [12] |
| NTO | Cys-modified AuNPs | Electrostatic interaction of NTO with AuNP@Cys and the formation of a Cu^2+^-coordination complex between particles to result in AuNPs agglomeration with a color change from red to violet | 0.25 mg L^–1^ | [13] |
| NTO | Unlabeled AuNPs | Anti-aggregation of AuNPs by NTO in the presence of MEL with a color change from blue to red | 1.53 attomol L^–1^ | [14] |
| Tetryl / NTO | Griess reagent in Fenton-like oxidation-driven by Cu_2_O@CuONC | The formation of a pink azo dye as a result of the reaction of the decomposition by-product nitrite, formed as a result of proposed Fenton-like oxidation catalyzed by Cu₂O@CuONC, with the Griess reagent. | Tetryl: 25.0 µg L^–1^  NTO: 2.5 µg L^–1^ | This work |

DCHA: dicyclohexylamine; PVC: polyvinyl chloride; DACH: diaminocyclohexane; TGA: thioglycolic acid; CTAB: cetyltrimethylammonium bromide; DDTC: diethyldithiocarbamate; EDA: ethylenediamine; TMB: 3,3',5,5'-tetramethylbenzidine; 4-ATP: 4-aminothiophenol; thiram: tetramethylthiuram disulfide; Cys: Cysteine; MEL: melamine; MNP: magnetite nanoparticles

Supplementary References

1. Sawicki CR, Scaringelli FP (1971) Colorimetric determination of nitrate after hydrazine reduction to nitrite. Microchem J 16:657-672

2. Wei H, Wang E (2008) Fe_3_O_4_ magnetic nanoparticles as peroxidase mimetics and their applications in H_2_O_2_ and glucose detection. Anal Chem 80:2250-2254.

3. Dai Lam T, Van Chat N, Bach VQ, Minh DB, Loi VD, Van Anh N (2014) Simultaneous degradation of 2, 4, 6-trinitrophenyl-N-methylnitramine (Tetryl) and hexahydro-1, 3, 5-trinitro-1, 3, 5 triazine (RDX) in polluted wastewater using some advanced oxidation processes. Journal of Industrial and Engineering Chemistry, 20(4), 1468-1475.

4. Yinon J (1999) Forensic and environmental detection of explosives. John Wiley & Sons.

5. Halasz A, Hawari J, Perreault NN (2018) New insights into the photochemical degradation of the insensitive unition formulation IMX-101 in water. Environ Sci Technol 52:589-596.

6. Şen N, Üzek U, Aksoy Ç, Bora T, Atakol O (2015) Identification of organic explosives which have different structures by LC-MS-MS. SDU J Scien 10:95-106.

7. DeTata D, Collins P, McKinley A (2013) A fast liquid chromatography quadrupole time-of-flight mass spectrometry (LC-QToF-MS) method for the identification of organic explosives and propellants. Forensic Sci Int 233:63-74.

8. Erçağ E, Üzer A, Apak R (2009) Selective spectrophotometric determination of TNT using a dicyclohexylamine-based colorimetric sensor. Talanta 78:772-780.

9. Ular N, Üzer A, Durmazel S, Ercag E, Apak R (2018) Diaminocyclohexane-functionalized/thioglycolic acid-modified gold nanoparticle-based colorimetric sensing of trinitrotoluene and tetryl. ACS Sens 3:2335-2342.

10. Özcan Ç, Üzer A, Durmazel S, Apak R (2019) Colorimetric sensing of nitroaromatic energetic materials using surfactant-stabilized and dithiocarbamate-functionalized gold nanoparticles. Anal Lett 52:2794-2808.

11. Yardımcı B, Koç ÖK, Üzer A, Hızal J, Apak R (2021) Ethylenediamine-bound magnetite nanoparticles as dual function colorimetric sensor having charge transfer and nanozyme activity for TNT and tetryl detection. Microchim Acta 188:1-12.

12. Demircioğlu T, Kaplan M, Tezgin E, Koç ÖK, Durmazel S, Üzer A, Apak R (2022) A sensitive colorimetric nanoprobe based on gold nanoparticles functionalized with thiram fungicide for determination of TNT and tetryl. Microchem J 176:107251.

13. Türkekul K, Üzer A, Can Z, Erçağ E, Apak R (2019) Colorimetric sensing of the insensitive energetic material 3-nitro-1, 2, 4-triazol-5-one (NTO) using l-cysteine stabilized gold nanoparticles and copper (II). Anal Lett 52: 2809-2821.

14. Durmazel S, Üzer A, Apak R (2022) Naked-eye detection of 3-nitro-1, 2, 4-triazole-5-one at sub-femtomolar levels with melamine and unlabeled Au nanoparticles. ACS Appl Nano Mater 5:5244-5257.
